# Cancer Chemoprevention: A Strategic Approach Using Phytochemicals

**DOI:** 10.3389/fphar.2021.809308

**Published:** 2022-01-13

**Authors:** Mohan Shankar G., Mundanattu Swetha, C K Keerthana, Tennyson P Rayginia, Ruby John Anto

**Affiliations:** ^1^ Division of Cancer Research, Rajiv Gandhi Centre for Biotechnology, Thiruvananthapuram, India; ^2^ Department of Surgery, Cedars-Sinai Medical Center, Los Angeles, CA, United States

**Keywords:** chemoprevention, chemopreventives, phytochemicals, phenolic compounds, tumor suppression, chemotherapeutics

## Abstract

Cancer chemoprevention approaches are aimed at preventing, delaying, or suppressing tumor incidence using synthetic or natural bioactive agents. Mechanistically, chemopreventive agents also aid in mitigating cancer development, either by impeding DNA damage or by blocking the division of premalignant cells with DNA damage. Several pre-clinical studies have substantiated the benefits of using various dietary components as chemopreventives in cancer therapy. The incessant rise in the number of cancer cases globally is an issue of major concern. The excessive toxicity and chemoresistance associated with conventional chemotherapies decrease the success rates of the existent chemotherapeutic regimen, which warrants the need for an efficient and safer alternative therapeutic approach. In this scenario, chemopreventive agents have been proven to be successful in protecting the high-risk populations from cancer, which further validates chemoprevention strategy as rational and promising. Clinical studies have shown the effectiveness of this approach in managing cancers of different origins. Phytochemicals, which constitute an appreciable proportion of currently used chemotherapeutic drugs, have been tested for their chemopreventive efficacy. This review primarily aims to highlight the efficacy of phytochemicals, currently being investigated globally as chemopreventives. The clinical relevance of chemoprevention, with special emphasis on the phytochemicals, curcumin, resveratrol, tryptanthrin, kaempferol, gingerol, emodin, quercetin genistein and epigallocatechingallate, which are potential candidates due to their ability to regulate multiple survival pathways without inducing toxicity, forms the crux of this review. The majority of these phytochemicals are polyphenols and flavanoids. We have analyzed how the key molecular targets of these chemopreventives potentially counteract the key drivers of chemoresistance, causing minimum toxicity to the body. An overview of the underlying mechanism of action of these phytochemicals in regulating the key players of cancer progression and tumor suppression is discussed in this review. A summary of the clinical trials on the important phytochemicals that emerge as chemopreventives is also incorporated. We elaborate on the pre-clinical and clinical observations, pharmacokinetics, mechanism of action, and molecular targets of some of these natural products. To summarize, the scope of this review comprises of the current status, limitations, and future directions of cancer chemoprevention, emphasizing the potency of phytochemicals as effective chemopreventives.

## Introduction

**FIGURE 1:**
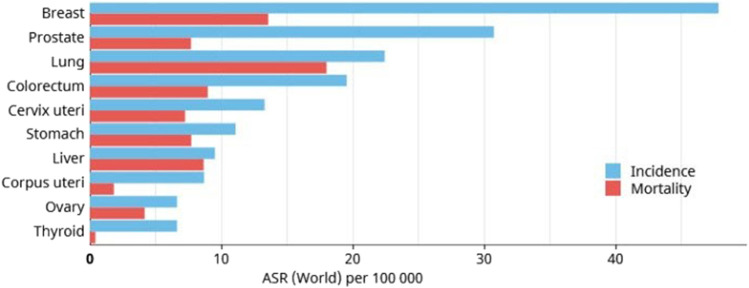
GLOBOCAN 2020 estimate of global incidence and mortality rates of different cancers, excluding non-melanoma skin cancer.

Cancer is one of the leading causes of mortality globally, accounting for almost 10 million deaths out of a total incidence of 19.3 million cases in 2020 (Sung et al., 2021), and is expected to increase over the next 2 decades. It is estimated that the global incidence of cancer could reach up to 28.4 million in 2040. Non-melanoma skin cancer records the highest incidence worldwide; however the mortality rate of this cancer is very low (Leiter and Garbe 2008; Lomas et al., 2012). According to GLOBOCAN 2020, breast cancer ranks at the top in terms of global incidence followed by prostate, lung and colorectal cancers (Sung et al., 2021). Lung cancer has the highest mortality rate followed by cancers of breast, prostate and liver. Figure 1 depicts the GLOBOCAN statistics regarding worldwide cancer mortality rates.

Cancer stems from intrinsic and/or extrinsic factors that derail the cellular signalling networks that maintain a balance between cell survival, proliferation and death. Research over many decades have shown that a single genetic alteration is insufficient to drive cancer as these are often countered by alternate mechanisms that leads to genomic repair or cell death. Hence, acquiring a lethal metastatic property requires widespread genetic and epigenetic modifications that endow the cell with the potential to undergo uncontrolled proliferation, invasion, and metastasis. This transformation happens over a long time frame, and is the reason for increased incidence rate in the elderly population. Though rapid strides have been made on research focussing on the aetiology and molecular underpinnings of cancer, these have not proved to be fruitful in improving therapeutic outcome in patients having late stage cancers. In addition, chemotherapeutic agents inflict severe side effects that curtail the quality of life of patients. Hence, it is imperative to design measures to mitigate cancer incidence or to impede the progression of benign neoplasm to advanced stage cancers. The idea of chemoprevention is gaining more popularity partly due to its success in lowering the incidence of cardiovascular diseases.

## Chemoprevention

**FIGURE 2:**
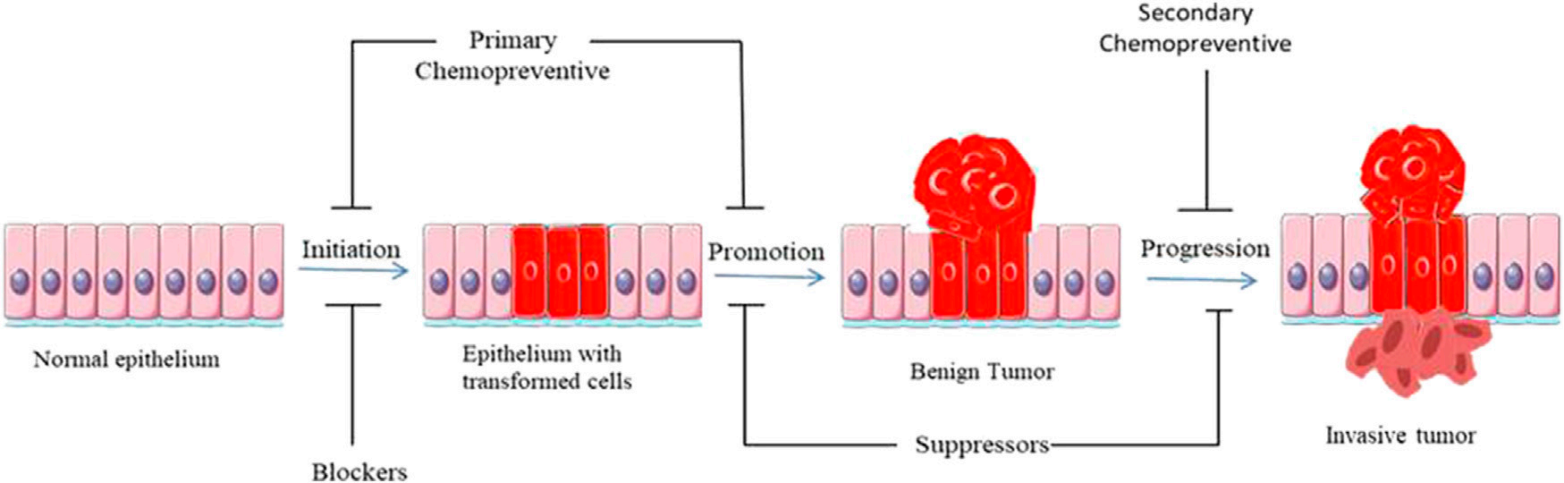
Diagrammatic representation of sequential progression of cancer, with special reference to the stages at which different classes of chemopreventives act.

Cancer chemoprevention is the use of natural and synthetic agents to suppress, prevent or delay tumorigenesis by blocking the initiation stage of carcinogenesis, or by curtailing the promotion stage wherein the initiated cells proliferate to give rise to a tumor. Figure 2 illustrates the sequential progression of cancer and the functional stages of different classes of chemopreventives. Compounds that block the initiation stage are generally termed as blocking agents, and the ones affecting the promotion stage are termed as suppressing agents (Chen and Kong, 2005). Blocking agents act in different ways like lowering the metabolic activation of pro-carcinogens into carcinogens, decreasing the level of reactive oxygen species (ROS), and induction of genomic repair pathways. Apart from blocking DNA-damage, initiation blockers may also exert tumor preventive effect by modulating epigenetic modification like hypermethylation of tumor suppressor genes. Suppressing agents may effectuate their chemopreventive efficacy by suppressing the signalling pathways that trigger cell survival and proliferation.

Depending on the stage at which they act, chemopreventives can be classified into primary, secondary and tertiary (Rather and Bhagat, 2018). Primary chemopreventives are aimed at suppressing tumor formation in a susceptible population. Secondary chemoprevention suppresses the transition of a tumor from benign to malignant phenotype. Tertiary chemoprevention lowers the risk of tumor recurrence following a successful surgical and/or chemotherapeutic intervention.

## Risk Factors in Carcinogenesis

Factors contributing to tumorigenesis have been categorized into two *viz*., intrinsic and extrinsic. Intrinsic factor refers to the spontaneous, random mutations in the genome that occurs during DNA replication. These mutations occur at different rates in different species. Extrinsic factors are those that occur due to exposure to biological and environmental agents like UV-radiation, chemicals and viruses. Aetiology of many cancers have been linked to extrinsic factors *viz.,* smoking and lung/oral cancer, viral infection and cervical cancer, exposure to asbestos and lung cancer, inflammation and colon cancer.

Table 1 summarizes the major risk factors involved in the development of various cancers.

**TABLE 1:** Extrinsic factors contributing to tumorigenesis.

Risk factor	Type	Cancer	References
UV and other ionizing radiation	Mutagen	skin cancer, leukemia, lung cancer	Bizzozero Jr et al. (1966), Veierød et al. (2003), Turner et al. (2011)
Cigarette smoke	Mutagen	Lung cancer	(Chapman et al., 2016); Guida et al. (2015)
Human papilloma virus	Mutagen	Head and neck cancer, Cancers of urogenital tract	(Cai et al., 2018, Sabatini and Chiocca 2020)
Hepatitis B virus	Mutagen	Liver Cancer	MacLachlan et al. (2015)
Asbestos	Mutagen	Pleural Mesothelioma	Tossavainen (2004)
Alcohol	Dietary factor	Breast cancer, liver cancer	Cancer (2002), Schütze et al. (2011)
Red Meat	Dietary factor	Breast cancer	Lo et al. (2020)

## Preclinical Observations

Clinical trials are majorly conducted based on preclinical evidences for the efficacy of the compound of interest, or epidemiological observations that give a strong correlation between cancer incidence and physiological level of a molecule. The pipeline for identifying prospective cancer chemopreventive agents starts from *in vitro* studies on cell lines. These studies assess the potency of a compound in inducing death of transformed cell lines, lowering its potency to migrate, invade, and revert the signalling framework to that of an untransformed cell. *In vitro* assays are followed by *in vivo* testing, which measures tumor incidence rate, size, number and grade of tumors. One of the best studied models for testing chemopreventive efficacy is the multi-stage skin carcinogenesis model wherein skin tumors are induced by application of DMBA for tumor initiation and PMA for tumor promotion. DMBA is also used to induce metastatic breast cancer in rats. Other potent carcinogens like Benzopyrene, Azoxymethane and Aflatoxin are commonly being used to induce tumors of lung, colon, and liver respectively. These models recapitulate the stage-wise progression of human cancers, and hence serve as reliable experimental systems to understand the biology of tumors, and to assess the tumor suppressive effects of novel compounds. These *in vitro*/*in vivo* studies have helped in understanding the molecular mechanisms by which these compounds exert chemopreventive efficacy. Here, we discuss results of pre-clinical studies on some of the compounds that were tested in clinical settings to assess their chemopreventive potential.

### Finasteride

Finasteride, a 5α-reductase inhibitor, was tested in multiple preclinical models of prostate cancer. In one study, the effect of finasteride in preventing prostate cancer induced by methyl nitrosourea/testosterone propionate was tested in Wistar rats. This study showed that rats orally administered with finasteride showed tumor incidence of only 10% while those in the control exhibited 64.3% incidence (Esmat et al., 2002). In another study the efficacy of finasteride in suppressing xenograft tumors were evaluated. Here, finasteride did not induce any change in growth of tumors from the metastatic cell line, LNCaP; however, it induced a significant increase in tumor burden in tumors induced by a combination of LNCaP and fibroblasts. Mechanistic studies showed that c-Jun, a component of the AP-1 transcription factor, plays the key role in driving tumor growth in finasteride administered animals (Niu et al., 2016).

### Dutasteride

Dutasteride is another 5α-reductase that was tested in clinical trials. In one study using LuCaP 35-generated xenograft models of prostate cancer, dutasteride was found to significantly reduce tumor growth. Dutasteride modulated the expression of genes involved in apoptotic, cytoskeletal remodeling, and cell cycle pathways (Schmidt et al., 2009). In another study, dutasteride induced apoptosis in androgen dependent prostate cancer cells but not in androgen independent cells. Dutasteride could also induce apoptosis in some but not all prostate cancer primary cultures. Here, the responsiveness of cells to dutasteride were dependent on the expression of antihuman α-methyl acyl-CoA racemase (AMACR) (Maria McCrohan et al., 2006).

### Pioglitazone

Pioglitozone, an inhibitor of peroxisome proliferator-activated receptor γ (PPARγ), was tested in preclinical models of lung cancer for assessing its chemopreventive efficacy. In a mouse model of lung cancer induced by injection of vinyl carbamate, pioglitazone could reduce tumor incidence by 64% when administered after 8 weeks post injection of carcinogen in *p53* wild type mice. In mice carrying a mutant p53 (*p53* ^*wt/Ala135Val*^), pioglitazone could suppress tumor incidence by 50% (Wang et al., 2010). In another study, pioglitazone was found to suppress benzo(a)pyrene induced lung cancer by 63%, and a combination of pioglitazone and budesonide could achieve 90% reduction in tumor burden (Fu et al., 2011).

### Epigallocatechin-3 Gallate

Epigallocatechin-3 gallate (EGCG) which is a key bioactive component in green tea extracts was found to induce reduction in tumor burden induced by 7,12-dimethylbenz(a) anthracene (DMBA) in Sprague-Dawley rats. Here, administration of EGCG delayed onset of mammary tumorigenesis, and reduced tumor invasivenesss (Kavanagh et al., 2001). In another study, EGCG was found to reduce the growth of MCF-7 xenografts in mice (Zan et al., 2019). Another study reported that physiological levels of EGCG caused growth inhibition in *P53* ^*WT*^MCF-7 cells while P53 mutant T47D cells were resistant to EGCG. Moreover, EGCG sensitized breast cancer cells to tamoxifen by upregulating estrogen receptor-α expression. EGCG also increased the expression of insulin-like growth factor receptor, and hence sensitized breast cancer cells to antibodies targeting these receptors (Zeng et al., 2014).

### Grape Seed Procyanidine Extract

Grape seed procyanidines have been shown to exhibit potent activity against lung cancer in both *in vitro* and *in vivo* conditions. Administration of different doses of procyanidins lead to a significant decrease in the growth of A549 and H1299 xenografts. This reduction in tumor burden was accompanied by an increase in the expression of insulin-like growth factor receptor in the tumor microenvironment (Akhtar et al., 2009). Later on, a study reported that the antineoplastic effect of procyanidines on lung cancer cells and the increased expression of insulin-like growth factor receptor is mediated by down-regulation of MicroRNA-19a/b. In this study, oral administration of leucoselectphytosome could mitigate growth of A549 xenografts (Mao et al., 2016).

### Celecoxib

Celecoxib, an inhibitor of COX-2, displays potent inhibitory activity against UV-induced skin carcinogenesis in murine models. Fischer et al. showed that inhibition of COX-2 could achieve dose-dependent reduction in UV-induced skin tumorigenesis. UV-induced synthesis of prostaglandins was significantly suppressed by the compound; however, tumors that constitutively expressed COX-2 did not show any growth reduction upon celecoxib treatment (Fischer et al., 1999). Another study showed that celecoxib could prevent the onset of new UV-induced tumors but the growth of pre-existing tumors remains unaffected by the inhibitor (Pentland et al., 1999).

### 2-phenethylisothiocynate

2-phenethylisothiocynate (PEITC), a cruciferous vegetable component, can inhibit cytochrome P450 enzymes which are involved in the conversion of carcinogens into their active forms (Nakajima et al., 2001). The compound also suppressed 4-(methyl nitrosamino)-1-(3-pyridyl)-1-butanone (NNK)-induced lung tumor by 50%, and could also suppress NNK-induced DNA methylation. However, PEITC could not suppress NNK-induced tumors in the liver or oral cavity (Morse et al., 1989).

## Clinical Trials

Similar to chemotherapeutic drug development, the testing of potential chemopreventive agents proceeds through many phases of studies on human subjects. Many clinical trials have been done in the past to assess the chemopreventive efficacy of compounds on the basis of epidemiological or pre-clinical data. Description of all the clinical trials is beyond the scope of the review. Hence, we detail some of the successful and failed clinical trials undertaken to assess chemopreventive potential of compounds, both natural and synthetic, on the most prevalent cancers.

### Breast Cancer

The Breast Cancer Prevention Trial (BCPT) was the first trial to show a significant positive result with chemoprevention. This study included 413,000 women at risk of breast cancer and showed that tamoxifen administration for 5 years lead to ∼50% reduction in breast cancer. However, tamoxifen administration caused higher incidence of endometrial cancer and thromboembolic events (Fisher et al., 1998). The efficacy of tamoxifen in reducing breast cancer incidence was further evaluated in another trial, IBIS-1, which showed similar effectiveness as seen in BCPT but the toxicity was shown to decline after 5 years post-termination of tamoxifen administration (Cuzick et al., 2007). In another study, tamoxifen was compared to raloxifene which showed that raloxifene was as effective as tamoxifen in reducing invasive breast cancer but were devoid of toxic effects. Later on, another study with increased median follow-up of 81 months showed that raloxifene is less effective than tamoxifen though it had a better safety profile. Tamoxifen was also found to be effective in preventing contralateral tumors (Cuzick et al., 2013). The effect of exemestane, an aromatase inhibitor, on breast cancer incidence was studied in a population of high risk women without breast cancer. Aromatase inhibitors block the synthesis of oestrogens from androgens. Exemestane significantly lowered overall incidence of breast cancer by 53% and suppressed the incidence of invasive breast cancer by 65% after a median follow-up of 3 years (Goss et al., 2011). A phase-2 double-blinded placebo-controlled clinical trial assessed the effect of green tea extract on mammographic density. In the treatment group, women consumed four decaffeinated GTE capsules having 1,315 mg of catechins for 12 months. The study showed that GTE capsules could reduce percent mammographic density in younger women but did not have any effect on older women. Further investigations will be necessary to assess the efficacy of GTE as a chemopreventive against breast cancer (Samavat et al., 2017). A study was undertaken to evaluate the effect of resveratrol, a bioactive compound present in berries and grape skin, on DNA-methylation pattern in women with high risk of breast cancer. The results show that the extent of methylation of RASSF-1α, a tumor suppressor gene, is inversely proportional to serum trans-resveratrol levels. The differential expression of RASSF-1α was found to be directly proportional to change in levels of prostaglandin E2 (Zhu et al., 2012).

### Prostate Cancer

The efficacy of finisteride, a 5α-reductase inhibitor, in lowering the incidence of prostate cancer was studied in a population of 18,882 men. After 7 years of administration of the drug, a reduction in incidence of prostate cancer was found though the chemopreventive efficacy was observed in low grade tumors (Thompson et al., 2003). Tumors with Gleason score of 7–10 were higher in finisteride group than in placebo group. Another trial studied the effect of Dutasteride on prostate cancer incidence. Similar to finisteride, a reduction of 22.8% was observed in prostate cancer in patients administrated with dutasteride and the effect was seen only in low grade tumors (Andriole et al., 2010). There were 12 tumors of Gleason score of 8–10 in dutasteride group compared to only one in the placebo group. In another study, the efficacy of selenium and vitamin E in preventing prostate cancer was assessed; however, the study was discontinued after an interim analysis, which indicated low chances of a positive result (Lippman et al., 2009). Another study showed that vitamin E administration could actually increase prostate cancer incidence (Klein et al., 2011). However, an independent study conducted by Heinonen et al showed that intake of α-tocopherol was correlated to reduced incidence of prostate cancer. Consumption of α-tocopherol lead to 32% decrease in prostate cancer incidence and 41% decrease in death (Heinonen et al., 1998). Two similar studies showed that selenium might exert chemopreventive efficacy against prostate cancer. Here, the effect was more pronounced in subjects with low basal selenium level in blood (Zhong et al., 2021).

### Lung Cancer

Chemoprevention trials on lung cancer have met with both success and failures. A phase-2 double-blind randomized placebo-controlled clinical trial of oral pioglitazone, a thiazolidinedione, was conducted on individuals at high risk of lung cancer incidence based on the observation that diabetes patients receiving thiazolidinediones have low lung cancer rates. The study was conducted on current or former smokers with sputum cytologic atypia or endobronchial dysplasia. Former smokers treated with pioglitazone showed mild improvement in the worst biopsy scores, and recorded a decreased Ki-67 labeling index of bronchial biposies. However, slight worsening was seen in current smokers administered with pioglitazone (Keith et al., 2019). The effectiveness of 2-phenethylisothiocynate (PEITC) in detoxifying the metabolites of 1,3-butadiene, a component of cigarette smoke, was studied in subjects who were smokers. 1,3-butadiene gets metabolized by cytochrome P450 enzymes into the active metabolites like 3,4-epoxy-1-butene (EB) which subsequently gets detoxified to mercapturic acids like MHBMA and DHMBA by GST enzymes. The clinical trial showed that oral ingestion of PEITC for 1 week increased urine MHBMA levels by 58.7 and 90% in GSTM1 and GSTT1 null subjects respectively, while it had negligible effect on other subjects (Boldry et al., 2020). This shows that PEITC could be a potential primary chemopreventive against tobacco smoke-induced cancers. A phase-1, open-label clinical trial was conducted to assess the chemopreventive efficacy, safety and tolerability of leucoselect phytosome (LP), a grape seed procyanidine extract (GSE) complexed with soy phospholipids. Bronchial biopsies were taken before and after 3 months of oral administration of LP. After 3 months, LP reduced Ki-67 labelling of bronchial biopsies by 55%, lowered serum oncomiRs, miR-19a,miR-19b,miR-106b, and it was well tolerated (Mao et al., 2021). Contrary to these positive results, some clinical trials have shown that some of the proposed chemopreventive agents could actually enhance tumor incidence. One of the earliest clinical trials on lung cancer chemoprevention was a placebo-controlled trial which enrolled 29,133 men administered with α-tocopherol and β-carotene either individually or in combination. Initially, the results showed an 18% increase in incidence of lung cancer and cardiovascular disease, and an 8% increased overall mortality for those on β-carotene (Albanes et al., 1995). Further analysis of the data showed that adverse effects were stronger in smokers and in men with moderate alcohol consumption. In theβ-Carotene and Retinol Efficacy Trial, men with occupational asbestos exposure or men and women who were current/former cigarette smokers were administered with either β-carotene plus retinyl palmitate or placebo. The study was discontinued as the subjects in the intervention group had more lung cancer and cardiovascular disease mortality rates (Omenn et al., 1996). In the β-carotene and retinol efficacy trial (CARET), administration of β-carotene to a population at high risk of lung cancer has led to 28% increase in lung cancer incidence (Group 1994). The α-tocopherol and β-carotene trial also reported a higher incidence in subjects who were administered with β-carotene. In another study that explored the effect of Non-steroidal anti-inflammatory drugs (NSAIDS) on incidence of small-cell lung cancer, it was observed that there was an increased risk of small-lung cancer incidence in subjects who had taken regular-strength aspirin (Brasky et al., 2012).

### Skin Cancer

A Phase-3 double blind, placebo-controlled clinical trial showed that oral ingestion of nicotinamide at a dose of 500 mg twice daily for 12 months decreased the incidence of new non-melanoma skin cancer (NMSC) by 23% in a high-risk population. The subjects recruited for the study had at least two non-melanoma skin cancer cases within 5 years prior to the study. No adverse effects were found in the nicotinamide treatment group during the 12-months period of the study (Chen et al., 2015). In another double-blind placebo-controlled randomized trial, the efficacy of celecoxib in lowering the incidence of actinic keratoses and NMSC was studied. Actinic keratosis is a precursor of cutaneous squamous cell carcinoma. The observed rate of transition of AK lesions into cutaneous squamous cell carcinoma is between 0.025 and 16% (Marks et al., 1988). Celecoxib at 200 mg was given to participants twice daily for 9 months. The results showed no difference in actinic keratosis incidence between control and placebo at 9 months post randomization; however, a decrease in NMSC incidence was observed 11 months after randomization in the group administered with celecoxib (Elmets et al., 2010). In another study, application of an ointment of curcumin on skin of patients with external cancerous lesions could induce symptomatic relief like reduction in smell and itching. Moreover, a reduction in lesion size and pain was seen in 10% of the patients (Kuttan et al., 1987). The efficacy of dl-α-tocopherol in suppressing potential transition of AKs to NMSC was studied in subjects with sun-damaged skin. Participants with AK lesions were administered with either placebo or 12.5% dl-α-tocopherol for 6 months. The levels of PCNA, p53, polyamines were assessed following treatment with the compound. The results showed no significant decrease in the number of AKs, expression of PCNA and p53; however, the level of polyamines was relatively low in the treated group. Though the authors of the study considers reduction of polyamines as a positive indicator of its chemopreventive potential in clinic, this effect could be outweighed by the unchanged expression status of p53 and PCNA (Foote et al., 2009). Hence, this warrants further trials using incidence of NMSC as the end point. The efficacy of 5-Fluorouracil in suppressing the incidence of basal cell carcinoma and squamous cell carcinoma requiring surgery was studied in veterans who are elderly (median age: 70) and had substantial exposure to Sun. Application of 5% 5-FU twice weekly for 2–4 weeks on face and ears substantially reduced the incidence of squamous cell carcinoma while no change was observed in the incidence of basal cell carcinoma during the first year of study. However, in the entire study period, there was no difference between treatment groups in time to first incidence of the carcinoma,keratinocyte, basal cell, or squamous cell carcinoma (Weinstock et al., 2018). Table.2 summarizes the clinical trials of some of the major chemopreventive molecules.

**TABLE 2:** Outcome from clinical trials using the phytochemicals as potential chemopreventives.

Molecule	Target cancer	Outcome in treatment arm	References	
Tamoxifen	Breast cancer	Reduction in breast cancer incidence but higher incidence of endometrial cancer	Fisher et al. (1998), Cuzick et al. (2013)	
Raloxifene	Breast cancer	Reduction in tumor incidence	Cuzick et al. (2013)	
Exemestane	Breast cancer	Reduction in tumor incidence	Goss et al. (2011)	
Finisteride	Prostate cancer	Reduction in incidence of low grade tumors but increased incidence of high grade tumors	Thompson et al. (2003)	
Dutasteride	Prostate cancer	Reduction in incidence of low grade tumors but increased incidence of high grade tumors	Andriole et al. (2010)	
Vitamin E	Prostate cancer	Reported both reduction and increase in tumor incidence	Klein et al. (2011), Heinonen et al. (1998)	
Selenium	Prostate cancer	Low tumor incidence in subjects with low basal level of selenium	Zhong et al. (2021)	
β-carotene	Lung cancer	Higher incidence of lung cancer	Albanes et al. (1995)	
Aspirin	Lung cancer	Higher incidence of lung cancer	Brasky et al. (2012)	
Nicotinamide	Skin cancer	Lowered the incidence of skin cancer	Chen et al. (2015)	
Celecoxib	Skin cancer	Lowered tumor incidence	Elmets et al. (2010)	
5-FU	Skin cancer	Lowered the incidence of cutaneous squamous cell carcinoma	Weinstock et al. (2018)	
Resveratrol	Breast cancer	Hypomethylation of RASSF-1α	Zhu et al. (2012)	
Curcumin	Skin cancer	Minor reduction in cancerous lesions; reduced smell and itching	Kuttan et al. (1987)	
Green tea extract	Breast cancer	Reduced mammographic density in younger women but not in older women	Samavat et al. (2017)	
2-phenethylisothiocynate	Lung cancer	Increased detoxification of metabolites of 1,3-butadiene	Boldry et al. (2020)	
Grape seed procyanidine extract	Lung cancer	Reduced Ki-67 labelling index of bronchial biopsies**;** lowered serum level of oncomiRs	Mao et al. (2021)	

Table 2 provides a brief description of the clinical trials conducted using the phytochemicals as chemopreventives and the outcome of the respective studies.

## Preclinical Evaluation of Natural Products as Prospective Chemopreventives

**FIGURE 3:**
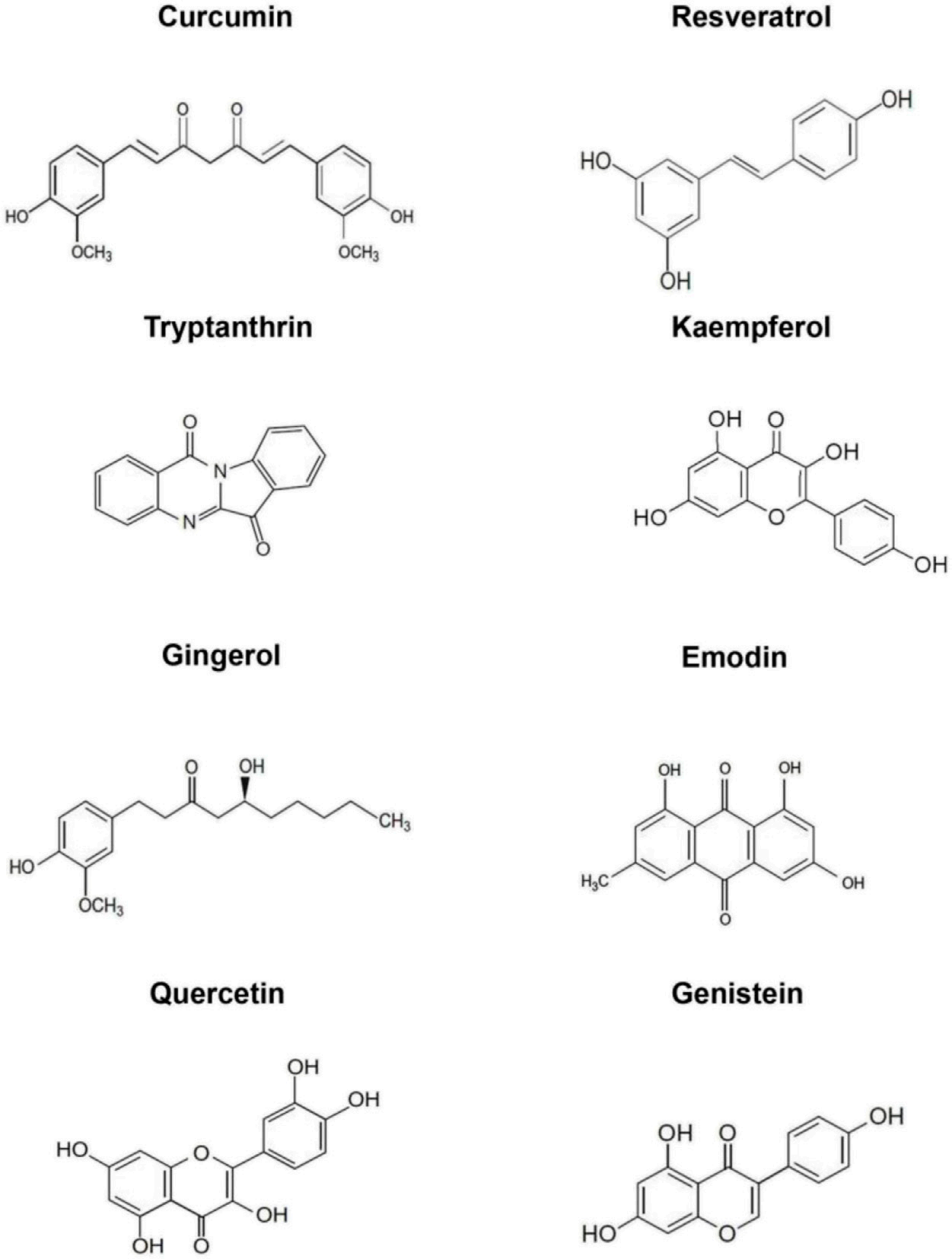
Chemical structures of the phytochemicals analysed as chemopreventives in the current study. i) Curcumin ii) Resveratrol (Nair et al., 2021) iii) Tryptanthrin (Shankar et al., 2015) iv) Kaempferol (Silva dos Santos et al., 2021) v) Gingerol vi) Emodin vii) Quercetin viii) Genistein (Nair et al., 2021).

Plants synthesize an array of secondary metabolites, which aid in fulfilling physiological functions as well as help in coping with exogenous constraints. Some of the major classes of secondary metabolites include polyphenols, flavanoids, alkaloids and anthraquinones. In the context of cancer, these phytochemicals have been extensively studied for their anti-oxidant, pro-apoptotic, anti-inflammatory, anti-angiogenic, anti-carcinogenic and anti-metastatic properties (Brglez Mojzer et al., 2016). The following section encompasses a brief account of various preclinical studies on the chemopreventive efficacy of some the predominant dietary phytochemicals. The chemical structures of the phytochemicals being discussed in this report are illustrated in Figure 3.

## Curcumin

Curcumin (diferuloylmethane), a polyphenol isolated from *Curcuma longa* is the most studied phytochemical as a chemopreventive. Curcumin displays a wide variety of pharmacological functions such as anti-inflammatory agent, anti-mutagenic agent, antineoplastic agent, hepatoprotective agent, nutraceutical, anti-microbial agent, anti-oxidant agent, and immunomodulator. It has been shown to have significant roles in prevention, treatment and chemo sensitization of cancer cells (Pavan et al., 2016). Studies have established the anti-proliferative, anti-angiogenic, anti-metastatic, and pro-apoptotic properties of curcumin (Anto et al., 2002; Puliyappadamba et al., 2010; Bava et al., 2011; Vinod et al., 2013).

We have reported that curcumin exhibits potential chemopreventive effect against nicotine-induced survival signalling in lung cancer cells. We found that it down-regulates multiple survival signals induced by nicotine in lung cancer cells irrespective of their p53 status (Puliyappadamba et al., 2010). Another study conducted in our laboratory revealed its chemopreventive efficacy against the lung carcinogenesis induced by B (a)P, a potential environmental carcinogen found in cigarette smoke and deep-fried food, in Swiss albino mice (Puliyappadamba et al., 2015). Curcumin treatment has a significant impact on improving the general health of colorectal cancer patients by enhancing expression of p53 molecules in tumour cells and also by promoting the apoptosis of tumour cells (He et al., 2011; Pricci et al., 2020). Curcumin intake causes the down-regulation of NF-kB, COX-2 and phosphorylated STAT3 in peripheral blood mononuclear cells from patients with pancreatic cancer (Dhillon et al., 2008). It has been reported that, daily curcumin intake leads to a significant and dose-dependent reduction in spontaneous ovarian cancer incidence and tumor growth in Hen ovarian cancer model indicating a significant role of curcumin as a chemopreventive strategy for ovarian cancer (Sahin et al., 2018). The administration of curcumin to paediatric patients with relapsed brain tumours undergoing chemotherapy increased their response compared with the controls (Wolff et al., 2012).

Resistance to chemo and radiotherapy is the major reason for cancer relapse. This arises due to the presence of a subpopulation of cancer cells, having self-renewal capabilities called Cancer Stem Cells (CSCs). Studies have confirmed that curcumin could inhibit the breast cancer stem cell population by downregulating the expression of stem cell genes Oct4, Sox2 and Nanog and also the Epithelial-Mesenchymal Transition (EMT) as observed by the down-regulation of mRNA levels of Vimentin, Fibronectin and β-catenin and up-regulation of mRNA levels of E-cadherin (Hu et al., 2019).

We have demonstrated that a combination of sub-optimal dose of 5-FU and curcumin elicits synergistic antitumor potential in murine models as evaluated by a reduction in the tumor-related parameters. Mechanistically, curcumin down-regulates 5-FU induced up-regulation of Thymidylate Synthase (TS), which is responsible for 5-FU chemoresistance (Vinod et al., 2013; Haritha et al., 2021). Another study from our lab reported that cervical cancer cells can be sensitized by curcumin to paclitaxel-induced apoptosis through down-regulation of NF-κB, Akt and Bcl2 (Bava et al., 2011). The chemopreventive agent curcumin also act as a potent radiosensitizer in human cervical tumor cells. Curcumin pre-treatment increased reactive oxygen species production and overactivation of the mitogen-activated protein kinase pathway in HeLa and SiHa cells when treated with Ionising Radiation (Javvadi et al., 2008).

However, therapeutic efficacy of curcumin is hindered to a certain extent by its low bioavailability (Toden and Goel 2017). To overcome this limitation, numerous curcumin analogues were developed, which include asymmetric curcuminoid analogues, curcumin analogue P1, curcumin analogue 3,5-Bis(2-pyridinylmethylidene)-4-piperidone (EF31), C-5 curcumin analogs etc. Asymmetric natural analogue of curcuminoid is shown to have anti-cancer activity by the presence of 4-hydroxy-3-methoxy benzene unit in it (Qiu et al., 2017). In Chronic Myeloid Leukemia and colon cancer cell line HCT116, C-5 Analog of curcumin shows the more cytotoxic effect by inhibiting the TNF-α-induced NF-κB activation than the curcumin (Allegra et al., 2017). Studies have reported that, purely aqueous PLGA nanoparticulate formulations of curcumin exhibit enhanced anticancer activity against human epithelial cervical cancer cells, HeLa (Nair et al., 2012). Our laboratory has demonstrated that, chitosan encapsulation enhances the bioavailability and tissue retention of curcumin and hence improves its efficacy in preventing Benzopyrene-induced lung carcinogenesis in Swiss albino mice (Vijayakurup et al., 2019).

Mechanism of action of curcumin mainly involves down-regulation of transcription factor NF-κB by inhibition of Notch signalling, which is involved in cell proliferation, apoptosis, maintenance of stem cell and their renewal. This results in a reduction in expression of NF-κB regulated genes, which includes Bcl-2, cyclin D1 and VEGF (O’riordan et al., 2005). Curcumin is a strong inhibitor of Protein Kinase C (PKC) and several oncogenes such as c-jun, c-fos, c-myc, NIK, MAPKs, ERK, ELK, PI3K, Akt and CDKs. Curcumin also inhibits of the Notch-1 downstream target Hes-1 in esophageal cancer cells. Hes-1 is an important notch signalling target and mediator (Subramaniam et al., 2012). The curcumin analog, 2-pyridylcyclohexanone has also been shown to decreases basal STAT3 phosphorylation and promotes apoptosis in esophageal cancer cell, ESCC cells (Wang et al., 2018).

Curcumin quenches free radicals, induces antioxidant enzymes (catalase, superoxide dismutase, glutathione peroxidase), and up-regulates antioxidative protein markers, Nrf2 and HO-1 that led to the suppression of cellular oxidative stress. In cancer cells, curcumin aggressively increases ROS that results in DNA damage and subsequently cancer cell death (Ak and Gülçin 2008).

Curcumin was found to suppress inflammatory cytokines such as IL-6, IL-8, granulocyte macrophage colony stimulating factor and TNF-α as well as IKKβ kinase in the saliva of HNSCC patients. Kim SG., et al., also suggested that IKKβ kinase could be a plausible biomarker for the detection of the effect of curcumin in head and neck cancer as curcumin inhibited IKKβ kinase activity and this resulted in the reduced expression of a number of cytokines (Kim et al., 2011).

Molecular docking studies further aids in identifying the role of curcumin in numerous signalling cascades involved in carcinogenesis and confirms the already suggested molecular mechansims responsible for the chemopreventive efficacy of curcumin. Using inverse molecular docking several proteins associated with cell proliferation and tumor formation namely, macrophage colony stimulating factor 1 receptor, aldo-keto reductase family 1 member C3, amiloride-sensitive amine oxidase and tyrosine-protein phosphatase non-receptor type 11 were identified as potential targets of curcumin. Curcumin was previously reported to inhibit the NFkB mediated activation of genes linked to cell survival and proliferation (Divya and Pillai 2006). Proteins such as MMP-2, NAD-dependent protein deacetylase sirtuin-2, core histone macro-H2A.1, NAD-dependent protein deacetylase sirtuin-1 and epidermal growth factor receptor were also revealed to be targets of curcumin, the binding of which regulates the activity of NF-kB (Furlan et al., 2018). These results provide a mechanistic explanation for the anticancer effects of curcumin. Targeting Phosphodiesterase 4 (PDE4) has been reported to be a potential therapeutic strategy against inflammatory disorders (I Sakkas et al., 2017). Studies suggest that curcumin may exhibit its anti-cancer property through the inhibition of PDE2 and PDE4 (Abusnina et al., 2015). Furlan et al. also gives evidences for the inhibitory effect of curcumin on PDE4 (Furlan and Bren 2021).

## Resveratrol

Resveratrol (3, 4′, 5-trihydroxystilbene), is a natural polyphenolic stilbene and common phytoalexin which is present mainly in grape skin, red wine, mulberries, blueberries, pistachio and peanuts. The abundant biological and pharmacological properties of resveratrol pave way to the wide-ranging therapeutic spectrum offered by the compound. Resveratrol has been shown to regulate oxidative stress, inflammation, apoptosis, and is also known to possess neuroprotective effects. Furthermore, it potentially regulates various cellular signaling events including immune cell regulation, cytokines/chemokines secretion, and also controls the expression of several immune-related genes. Resveratrol is capable of modulating various cellular events such as apoptosis, autophagy, cell cycle, inflammation, invasion and metastasis, which collectively contribute to its chemopreventive efficacy.

As a chemopreventive agent, resveratrol influences all the major stages of carcinogenesis. Being polyphenolic in nature, it displays strong antioxidant activity and exercises control over multiple molecular events. Resveratrol causes the activation of tumor suppressor genes and inhibition of oncogenes that are crucial to carcinogenesis. Resveratrol has been shown to possess pro-apoptotic role and is known to decrease the viability and mitotic index of a number of cancer cell lines. Several studies have documented the excellent chemopreventive effects of resveratrol in various cancer types including oral, colorectal, prostate and breast cancer. Previous studies have demonstrated that resveratrol potentially suppressed the transcription and translation of E6 and E7, through induction of apoptosis and by causing G1/S phase transition arrest (Sun et al., 2021), thereby inhibiting cervical cancer under both *in vitro* and *in vivo* conditions. Resveratrol operates through various signalling pathways such as STAT3, AKT/mTOR AMPK, IGFR and Wnt pathways (Lohse et al., 2018). Amin et al., investigated the *in vitro* and *in vivo* antitumor effects of a combination of epigallocatechin gallate (EGCG) and resveratrol, and they found that their combination at low doses exhibited a synergistic growth inhibition in head and neck cancer, both *in vitro and in vivo*. Furthermore, the mechanistic studies unravelled that the combination inhibited AKT-mTOR signaling and the over-expression of constitutively active AKT protected cells from apoptosis induced by the combination of EGCG and resveratrol (Amin et al., 2021). Numerous studies have established SIRT1 to be one of the key targets of resveratrol. An up-regulation of SIRT1 mandates the chemopreventive effects of resveratrol in colorectal cancer cells (Buhrmann et al., 2016). Resveratrol potentially down-regulates NF-κB phosphorylation which consequently restricts tumor invasion and metastasis. Previous reports have mentioned the significant role of NF-ĸB in manifesting SIRT1-dependent anticancer activity of resveratrol (Bourguignon et al., 2009). MALAT1 is a key driver in the progression of multiple cancers including renal, cervical, liver, osteosarcoma, etc. Resveratrol hinders the invasion and metastasis of colorectal cell lines via MALAT-1-mediated Wnt/β-catenin signaling pathway (Ji et al., 2013). Studies involving mouse mammary organ culture model have revealed that resveratrol prevents the transcriptional activation of cytochrome P-450 1A1. Resveratrol is also known to cause blockade of G1/S phase transition in a number of cell lines. Resveratrol has been proven to inhibit the expression of COX-1/2. Furthermore, it downregulates TPA-induced activation of protein kinase C and AP-1-mediated gene expression in mammary epithelial cells (Bhat and Pezzuto, 2002). Resveratrol targets a wide array of molecules including ERK 1, PI3K, ERα/β, AMPK, AKT1, STAT3, FAS, COX1/2, p53, NF-κB, IKKB, p38, JNK 1. Studies on the effect of resveratrol on three mutagens namely, aflatoxin B1, 2-amino-3-methylimidazo (4,5-f) quinoline and N-nitroso-N-methyl urea have established that resveratrol significantly decreased the mutagenicity of all three mutagens (Langová et al., 2005). In spite of its remarkable cancer chemoprevention properties, the low oral bioavailability of resveratrol has often impeded its translation to *in vivo* effects. Earlier reports indicate that while the oral administration of resveratrol effectively inhibited colorectal carcinogenesis, it failed to protect mice from chemical-induced lung carcinogenesis. Therefore, Monteillier et al., attempted intranasal administration of resveratrol and they observed that this method successfully prevented lung cancer in A/J mice. This proves to be an effective solution to overcome the issue of low oral bioavailability of the compound (Monteillier et al., 2018).

Besides being an excellent chemopreventive, reseveratrol also functions as an effective chemosensitizing agent. Our studies have established that docetaxel and resveratrol elicit a synergistic response against Her-2 over-expressing breast cancer cells. We have also documented that HER-2–Akt signaling axis plays a significant role in regulating the synergistic effect of docetaxel and resveratrol (Vinod et al., 2015).

Multiple studies have elucidated the strong anti-oxidant potential of resveratrol. The compound modulates NF-κB pathway and confers protection against myocardial ischemic injury and aflatoxin induced hepatocellular carcinoma (Rawat et al., 2021; Ro et al., 2021).

Molecular docking studies identified the proteins NTMT1, LSD1 and BIRC4 as potential targets of resveratrol, the inhibition of which could contribute to the chemopreventive effects of resveratrol (Kores et al., 2019).

## Tryptanthrin

Tryptanthrin, an indoloqinazoline alkaloid, was isolated initially by sublimation of natural indigo. Following this, the compound was isolated from other natural sources like *Isatis, polygonim* and *Wrightia* species. The compound exhibits antimicrobial, anti-inflammatory, antiprotozoan and antiparasitic activity. Tryptanthrin was also found to be effective against intestinal disorders and allergy.

Multiple studies have demonstrated the chemopreventive efficacy of tryptanthrin. The efficacy of tryptanthrin as a primary chemopreventive was first studied in animal models of intestinal tumorigenesis. In this study, the effect of tryptanthrin in suppressing azoxymethane-induced intestinal tumors in F344 rats was compared to that of a crude ethyl acetate extract of *Polygonumtinctorium Lour*. Animals were administered with either 15 mg/kg of Azoxymethane for 3 weeks to induce atypical crypt foci or 7.5 mg/kg of Azoxymethane for 10 weeks to induce intestinal tumors. It was observed that in the short-term experiment, the incidence of atypical crypts was significantly lower in both the treatment groups when compared to the control. Similarly, in the long term study, the treatment groups had low intestinal tumor burden when compared to the control (Koya-Miyata et al., 2001).

We have demonstrated the potency of tryptranthrin in suppressing the promotion stage of skin carcinogenesis. Here, tryptanthrin at a dose of 1 mg when applied before each application of PMA on DMBA-initiated skin could suppress tumor burden in terms of both tumor size and number. Though we have observed 100% incidence in the treatment group at the end of the experiment, there was significant delay in tumor incidence and this could be many years when extrapolated in a clinical setting. Mechanistic evaluation showed its ability to suppress the proliferation of hair-follicle cells and downregulated key pro tumorigenic signaling pathways *viz*., MAPK, β-catenin. Tryptanthrin could also accomplish reduction in PMA-induced immune cell infiltration into the epidermis. Further, we demonstrated that the compound could exert anti-cancer activity comparable to 5-FU against the epidermoid cancer cell line, A431 (Shankar G et al., 2020).

The efficacy of tryptanthrin as an anticancer compound has also been studied. Tryptanthrin exhibits appreciable activity against cancers of different origins like blood, lung, colon etc. Tryptanthrin at low concentrations induced the differentiation of monocytic and promyelocyticleukemia cells while it induces caspase-mediated apoptosis at higher concentrations (Kimoto et al., 2001). Tryptanthrin was found to exhibit moderate anti-cancer effect against lung cancer cells (Yang et al., 2013). The compound also exerts significant anticancer effect against the neuroblastoma cell line, LA-N-1 by attenuating the expression of N-myc (Liao and Leung 2013).

Tryptanthrin affects a wide variety of cellular processes that are implicated in many diseases. Tryptanthrin is known to exert a protective effect against hepatocyte stress by affecting ERK2 and Nrf2 pathways (Moon et al., 2014). Similarly, *Indigo naturalis*, in which tryptanthrin is one of the bioactive compounds, is known to protect keratinocytes from oxidative stress, by abrogating intracellular ROS formation (Lin et al., 2013).

Tryptanthrin is known to inhibit the activity of COX-2 with the inhibitory potential comparable to standard COX-2 inhibitors like NS-398 and nimesulide (Danz et al., 2001). In concordance to this observation, another study reported that tryptanthrin can inhibit inflammation by suppressing the formation of prostaglandins and leukotrienes (Danz et al., 2002). Tryptanthrin was also found to exert inhibitory activity against 5-LOX, another mediator of inflammation (Danz et al., 2002). In another study, tryptanthrin was found to exert anti-inflammatory activity in murine macrophage-like RAW 264.7 cells by lowering the expression of iNOS through down-regulation of NF-κB (Ishihara et al., 2000).

Tryptanthrin is reported to be an inhibitor of angiogenesis, a vital process in cancer progression. In this study, tryptanthrin was found to inhibit the proliferation, migration and tube formation of human microvascular endothelial cells (HMEC-1) in a concentration dependent manner. The compound was found to suppress angiogenesis by inhibitingVEGFR2-ERK1/2 signaling (Liao et al., 2013). The study showed that VEGFR2 is a direct molecular target of tryptanthrin.

## Derivatives of Tryptanthrin

Multiple structural derivatives of tryptanthrin and their nanoformulations have been synthesized and their activities against cancer were assessed by many researchers. These studies reveal the enhancement of the activity of tryptanthrin after structural modification.

A bromo analogue of tryptanthrin (TBr) was found to induce apoptosis in leukemia cell lines. Treatment of the leukemia cell line, HL-60 with TBr caused the inactivation of STAT-3 through a ubiquitin dependent mechanism (Pathania et al., 2014). In another study the copper derivative of tryptanthrin (Try-Cu) and bromotryptanthrin (BrTry-Cu) was tested for their cytotoxic activity against four cancer cell lines, BEL-7402, T-24, MGC80-3 and Hep-G2 (Qin et al., 2018). It was observed that Try-Cu exhibited appreciable anti-cancer activity against all for cancer cells while being non-toxic to the normal cell line, HL-7702. Another study revealed the potential of platinum complexes of tryptanthrin in exerting anti-cancer activity against the human bladder cancer cell line, T-24 without affecting the normal cells. Benzo(b)tryptanthrin, the benzo-annulated derivative of tryptanthrin exerts cytotoxicity in several human cancer cells. Benzo(b)tryptanthrin induced apoptosis through the activation of caspase-3 in the colon cancer cell line, HCT15. Benzo(b)tryptanthrin also reversed adriamycin resistance in breast cancer cells by down-regulation of multidrug resistance protein 1 (MDR1). Interestingly, this effect was much better than that displayed by tryptanthrin (Jun et al., 2015).

A study on the pharmacokinetic properties of tryptanthrin showed that oral administration of 80 mg/kg of the compound to mice models yielded maximum plasma concentration of 3.13 µg/ml, and the maximum concentration was reached within 2.5 h (Zhang et al., 2017). Attempts have been made for improving the therapeutic efficiency of tryptanthrin by improving its pharmacokinetic properties. Fang et al., encapsulated tryptanthrin in various nanoparticles like solid lipid nanoparticles, nanostructured lipid carriers and lipid emulsions. Among the three systems, the release rate of tryptanthrin was found to be maximum in nanostructured lipid carriers. The cytotoxicity against MCF-7 cells were significantly increased upon treatment with nanoparticle encapsulated tryptanthrin, suggesting that encapsulation of the compound in nanoparticles can improve drug delivery and facilitate the sustained release of the compound to the cells (Fang et al., 2011).

## Kaempferol

Kaempferol [3,4′,5,7-tetrahydroxyflavone-(MW: 286.2 g/mol)] is a major flavonoid aglycone found in many natural products. Kaempferol displays several pharmacological properties, such as antimicrobial, anti-inflammatory, antioxidant, antitumor, cardioprotective, neuroprotective, and antidiabetic activities. We have done an overview of its major applications in the field of cancer therapy.

Kaempferol has been shown to effectively inhibits the growth of breast cancer cell lines (VM7Luc4E2, MDA- MB-231, MCF-7) (Azevedo et al., 2015). In the highly invasive breast cancer cell line, MDA-MB-231, kaempferol did significantly inhibit MMP-3 protein activity in a dose-dependent manner, which accounts for its anti-metastasis property (Ferraz da Costa et al., 2020). It has been reported that kaempferol inhibits both growth and migration of glioma cells, *in vitro* (Jeong et al., 2009). Kaempferol activates the IRE1-JNK-CHOP signaling from cytosol to nucleus, and G9a inhibition, which activates autophagic cell death in gastric cancer cells (Kim et al., 2018). Our laboratory has characterized kaempferide, a methyl derivative of kaempferol, isolated from the plant, *Chromoleana odorata.* Among the cancer cell lines that were screened against the compound, the cervical cancer cell line, HeLa was the most sensitive. Kaempferide is pharmacologically safe in murine models and exhibits excellent anti-tumor efficacy *in vivo* (Nath et al., 2015).

Kaempferol significantly inhibits the proliferation of human hepatic cancer cell lines (HepG2, SK-Hep-1, Huh7) (Mylonis et al., 2010) and human colorectal cancer cell lines (HCT116, HT-29, HCT-15, LS174-R colon, and SW480). Kaempferol treatment increases membrane-bound FAS ligand levels, decreases intact caspase-8 and Bid, and increases cleavage of caspase-8 in human colon cancer cells indicating that kaempferol-induced apoptosis is associated with the activation of cell surface death receptors and the mitochondrial pathway (Lee et al., 2014). Experimental studies combining kaempferol with 5-Fluorouracil in LS174-R cells has revealed interesting antiproliferative effects (Riahi-Chebbi et al., 2019). Experiments using human ovarian cancer cell lines (A2780/CP70, A2780/wt, SKOV-3, OVCAR-3) have shown that kaempferol could inhibit tumor growth, proliferation, and angiogenesis by decreasing vascular endothelial growth factor (VEGF) expression (Luo et al., 2009).

Kaempferol has the capacity to decrease the production of free radicals and reactive oxygen species (ROS). ROS production inhibition can reverse malignant cancer cell phenotype. Kaempferol can control the cancer through its antioxidative property by inhibiting the NF-κB pathway and up-regulating the Nrf2 transcriptional pathway (Saw et al., 2014).

Kaempferol appears to inhibit VEGF expression and angiogenesis through an ERK-NFκB-cMyc-p21 pathway. Kaempferol administration has been shown to discourage ERK phosphorylation as well as NFkB and c-Myc expression, the reduction of which promotes p21 expression. p21 is a tumor suppressor protein known to antagonize VEGF secretion (Luo et al., 2012). A diet high in flavanols, especially kaempferol, has been found to correlate with reduced serum interleukin-6 levels, an inflammatory cytokine. In HEK 293 cells, kaempferol blocked both TNF-induced IL-8 promoter activation, but also IL-8 gene expression. IL-8 has been found to be a potent enhancer of angiogenesis (Bobe et al., 2010).

Studies on the *in vitro* and *in vivo* pharmacokinetics of kaempferol commonly ingested as high polarity glycosides has revealed that this polyphenol is poorly absorbed compared to the aglycones with intermediate polarity (Boadi et al., 2020). Kaempferol shows very low bioavailability of approximately 2%. Nano research had also been conducted focusing on enhancing the bioavailability of kaempferol specifically. The PEO-PPO-PEO (Poly (ethylene oxide)-poly (propylene oxide)-poly (ethylene oxide) formulation and the PLGA (Poly (DL-lactic acid co-glycolic acid) encapsulated kaempferol have been shown to improve efficacy of the compound in preferentially killing malignant cells (Chen and Chen, 2013).

Moreover, the inhibitory effect of kaempferol on NF-κB was revealed in a molecular docking study where kaempferol was compared with MG-132, a known inhibitor of NF-κB. In silico calculations suggest that kaempferol inhibits the DNA binding pf NF-κB by intercalating into DNA thus accounting for its anti-inflammatory and anticancer activities (Fiedler et al., 1998; Kadioglu et al., 2015).

## Gingerol

Gingerol [5-hydroxy-1-(4-hydroxy-3-methoxyphenyl) decan-3-one], is an aromatic ketone, and the most abundant constituent of the fresh roots and rhizomes of ginger plant, Zingiber officinale (Zhang et al., 2021). Gingerol is responsible for the strong pungency of ginger and is one of the major active components of the plant. Though several derivatives of gingerol are present in ginger, 6-gingerol is the most abundant among them. Gingerol has a wide array of pharmacologic effects. It is highly effective against chemotherapy related nausea and vomiting. Gingerol possesses anti-cancer, antioxidant, anti-angiogenic, anti-atherosclerotic anti-spasmodic and hepatoprotective potentials. 6-Gingerol possesses remarkable anticancer potential and it affects a variety of biological pathways involved in apoptosis, cell cycle regulation, cytotoxic activity, and inhibition of angiogenesis.

Studies conducted in our laboratory have revealed that (6)-gingerol induces caspase-dependent apoptosis in colon cancer cells and prevents PMA-induced proliferation through inhibition of MAPK/AP-1 signaling. The underlying mechanism was found to be the down-regulation of PMA- induced phosphorylation of ERK1/2 and JNK MAP kinases and activation of AP-1 transcription factor. However, it showed only little effects on phosphorylation of p38 MAP kinase and activation of NF-κB (Radhakrishnan et al., 2014). Nigam et al., have reported that (6)-gingerol mediated induction of apoptosis is associated with the modulation of p53 and involvement of mitochondrial signaling pathway in B [a]P-induced mouse skin tumorigenesis (Nigam et al., 2010). (6)-gingerol has also been proven to impede hamster buccal pouch carcinogenesis associated with chemically-induced inflammation and cell proliferation via the modulation of Nrf2 signaling (Sun et al., 2021). Gingerol elicits its protective activity through different mechanisms and cell signaling pathways, of which, MAPK, NF-κB, Wnt/β-catenin, Nrf2/ARE, TGF-β1/Smad3, and ERK/CREB are prominent (Yahyazadeh et al., 2021).

(10)-Gingerol, a derivative of gingerol has been shown to improve the anti-cancer efficacy of doxorubicin and ameliorate the side effects caused by the drug in triple negative breast cancer models (Martin et al., 2020).

Gingerol and shogaol, and other structurally-related substances inhibit the biosynthesis of prostaglandin and leukotriene. They are also capable of inhibiting the synthesis of pro-inflammatory cytokines such as IL-1, TNF-α, and IL-8 (Shukla and Singh 2007; Ray et al., 2015).

Molecular docking studies between four ginger ligands, namely, 6-gingerol, 8-gingerol, 10- gingerol, 6-shogaol and identified cancer targets such as EGFR, C-Met, PI3K, COX-2, NF-kB, and AP-1 suggests that 6-gingerol is more effective as an anticancer phytocompound among ginger ligands (Kumara et al., 2017).

It is well known that (6)-gingerol efficiently scavenges chemical carcinogens, especially those belonging to the epoxy type. The pharmacokinetics underlying adduct-formation by (6)-gingerol has been investigated (Furlan and Bren 2020). The study focused on the changes in the activation free energy of the rate-limiting step of the alkylation reactions of (6)-gingerol with nine epoxy type chemical carcinogens. The activation barrier i.e., ΔG^⧧^, for the reaction between natural scavengers and chemical carcinogen is much lower than that of the competing reaction between the chemical carcinogen and nucleophilic DNA base, guanine. Hence, (6)-gingerol confers protection against carcinogen-mediated DNA alkylation and prevents initiation of cancer by virtue of its lower activation barrier and the resultant faster reaction rate *via SN2* reaction mechanism. An independent study has reported the pharmacokinetics of various derivatives of gingerol using HRMS analytical method which was followed by oral administration of ginger extract in rats to assess the distribution of gingerol derivatives in tissues. Furthermore, they quantified the concentration of 6-gingerol, 6-shogaol, 8-gingerol, 8-shogaol, 10-gingerol and 10-shogaol in the plasma and tissues of rats. The results illustrated that 6-gingerol, 6-shogaol, 8-gingerol, 8-shogaol, and 10-gingerol are rapidly absorbed into the circulatory system, but, 10-shogaol is poorly absorbed in comparison to the other compounds upon administration of ginger extract orally. It was also observed that 6-shogaol failed to penetrate the blood–brain barrier and enter the brain (Li et al., 2019). However, the pharmacokinetics of gingerol and its derivatives needs to be explored in further detail.

## Emodin

Emodin (1, 3, 8-trihydroxy-6-methylanthraquinone) is a naturally occurring anthraquinone present in the roots and barks of plants such as *Cassia obtusifolia, Fallopia japonica, Polygonum cuspidatum*, and *Rheum palmatum* (Dong et al., 2016). It is also present in certain species of moulds and lichens. Emodin is an active constituent of numerous Chinese medicinal herbs. Emodin is a well- known tyrosine kinase inhibitor and displays an inhibitory effect on mammalian cell cycle modulation in specific oncogene over-expressed cells (Sakalli-Tecim et al., 2021). Emodin is known to inhibit angiogenesis and metastasis processes which make it a promising candidate for chemoprevention.

Several studies have demonstrated the chemopreventive potential of emodin. The anti-tumor promoting effect of emodin was elucidated using two-stage chemically induced carcinogenesis models of skin tumor in mice (Koyama et al., 2002). Huang et al., have demonstrated emodin-mediated inhibition of HSC5 and MDA-MB-231 cell invasion by inhibiting AP-1 and NF-κB signaling pathways (Huang et al., 2004). Previous reports illustrate the ability of emodin to directly target androgen receptor and in turn suppress prostate cancer cell growth *in vitro* and prolong the survival of C3 (1)/SV40 transgenic mice *in vivo*. It is speculated that emodin treatment represses the androgen-dependent transactivation of androgen receptor (AR) by inhibiting nuclear translocation of AR (Cha et al., 2005). Studies by Shimpo *et al.*, have revealed that dietary administration of low dose of aloe emodin, a derivative of emodin exerts chemopreventive effects against development of colorectal tumor in mice by reducing cell proliferation in colorectal mucosa (Shimpo et al., 2014). Emodin at concentrations of 10–20 μM has been reported to trigger apoptosis of IMR-32 cells *via* an apoptotic signaling cascade which sequentially involves ROS, Ca^2+^, NO, p53, caspase-9 and caspase-3 (Huang et al., 2013). Epstein-Barr virus (EBV) lytic replication plays an important role in the pathogenesis of nasopharyngeal carcinoma. Emodin inhibits the tumorigenic properties induced by repeated EBV reactivation, which encompasses micronucleus formation, cell proliferation, migration, and matrigel invasiveness and repression of tumor growth in mice which is induced *via* EBV activation (Wu et al., 2019). Previous studies illustrate that emodin potentiates apoptosis in a p53-dependent manner in SK-HEP-1, PLC/PRF/5, and HepG2/C3A cells (Shieh et al., 2004). Emodin is believed to function as a Janus-activated kinase 2 inhibitor, which accounts for its cytotoxic effects against multiple myeloma (Muto et al., 2007). Emodin has been reported to prevent lipid raft coalescence in HepG2 cells, impede the gathering of integrin in HeLa cells and restrict the formation of focal adhesion complex (FAC) in MDA-MB-231 cell lines (Huang et al., 2006). There is documented evidence that emodin treatment stimulates Cyt- c release and activates caspase-2, -3, and -9. Emodin treatment in A549 cells resulted in the inactivation of AKT and ERK and formation of ROS, Further, it disrupted the mitochondrial membrane potential and reduced the levels of mitochondrial Bcl-2 and increased the mitochondrial Bax levels (Akkol et al., 2021). Studies conducted in our laboratory has proved that emodin-induces caspase-dependent apoptosis in human cervical cancer cells presumably through the mitochondrial pathway (Srinivas et al., 2003).

Emodin chemosensitizes a wide spectrum of chemotherapeutic drugs including paclitaxel, platinum drugs, 5-FU and As2O3 (Vinod et al., 2013). Several reports have elucidated the chemosensitizing efficacy of emodin in various types of cancer which include melanoma, pancreatic, ovarian, renal, cervical, colorectal, prostrate and lung cancer.

Reversal of multidrug resistance, induction of autophagy and apoptosis are the major pharmacological roles of anthraquinones in cancer cells (Liu et al., 2020). Guo et al., have documented that emodin mediated inhibition of MDR1/P-glycoprotein and expression of MRPs alleviates gemcitabine resistance in pancreatic cancer (Guo et al., 2020).

Previous studies have speculated that the immunosuppressive effect of emodin is mediated through hydrogen peroxide and regulated by the by products of arachidonic acid metabolism (Huang et al., 1992).

Emodin treatment decreased the mutagenicity of benzo (a)pyrene [B (a)P], 2-amino-3-methylimidazo (4,5-f)quinoline (IQ) and 3-amino-1-methyl-5H-pyrido (4,3-b) indole (Trp-P-2). in a dose-dependent manner in *Salmonella typhimurium* TA98. This was achieved through emodin-mediated direct inhibition of the hepatic microsomal activation (Lee and Tsai, 1991). Previous studies have attributed the poor oral bioavailability of emodin to its glucuronidation metabolism. Shia et al., have studied the *in vivo* levels of emodin after intagastric administration in rats and they observed that the detected *in vivo* emodin levels remained extremely low (Shia et al., 2010). Another study revealed that piperine could considerably increase the Cmax and area under concentration-time curve (AUC) of emodin and cause a simultaneous decrease in the AUC and Cmax of emodin glucuronide (Di et al., 2015).

## Quercetin

Quercetin, also known as 3, 3, 4, 5, 7—pentahydroxyflavone, is a flavonoid generally present in several fruits, vegetables, leaves, seeds and grains, where it is conjugated with residual sugars such as glucose, rutinose, or xylose to form quercetin glycosides. Several reports suggest that quercetin can induce cell cycle arrest and apoptosis by virtue of its antioxidant (Robaszkiewicz et al., 2007), anti-inflammatory and immune protective effects (Nair et al., 2002). Quercetin and its derivatives being naturally occurring phytochemicals with promising bioactive effects, its intake via diet or food supplements might ensure a protective effect.

*In vitro* studies conducted by Senthilkumar et al., presents evidences of an inhibitory effect of quercetin on androgen independent prostate cancer, where quercetin was found to modulate the expression of the components of IGF system leading to apoptosis (Senthilkumar et al., 2010). Quercetin was also reported to significantly reduce the level of VEGF-3 in PC-3 cells, indicating its antiangiogenic properties (Pratheeshkumar et al., 2012).

Chemopreventive effect of quercetin in *in vivo* model of prostate cancer has been demonstrated for the first time by Sharmila et al., in Sprague Dawley rats. Animals were given a periodical administration of the carcinogen N-nitroso-N-methyl urea (MNU) and hormone testosterone with a simultaneous supplementation of quercetin (Sharmila et al., 2014). The study revealed that quercetin supplementation decreases the expression of IGF-1R, by reducing pAkt, Raf-1 and pMEK protein expressions in comparison with the cancer induced rats. Over expression of IGF-1/IGF-1R has been found to result in the initiation of prostate cancer (Cox et al., 2009) with the PI3/Akt and Ras/Raf/MEK/MAPK being the major pathways associated with the activation of the same (Ozkan, 2011).

Quercetin has previously been reported to affect the signal transduction pathways involved in the process of carcinogenesis which eventually result in the induction of apoptosis and inhibition of cell proliferation (Sun et al., 2010). Similar studies were conducted by other groups in DMBA -treated hamsters with the aim of investigating the chemopreventive efficacy of quercetin on oral squamous cell carcinoma (OSCC) and its mechanism of action. The study has reported that the animals in the groups that received medium (25 mg/kg) to high (50 mg/kg) doses of quercetin showed no tumor development (Zhang et al., 2017). The study suggests that the chemopreventive effect displayed by quercetin in the DMBA-induced carcinogenesis model could be on account of suppression of NF-κB pathway by quercetin, followed by the modulation in the expression of NF-κB target genes Bax and Bcl-2, which led to apoptosis and tumor regression.

Oxidative stress plays a central role in cancer development and progression as it promotes damage to proteins, lipids, membranes and DNA alike (Khandrika et al., 2009). The anticancer potential of quercetin can be attributed to various mechanisms, such as the induction of cell cycle arrest and/or apoptosis, as well as its antioxidant properties. Quercetin has been shown to exhibit its antioxidant activity through its regulatory effect on glutathione (GSH), enzymatic activity, signal transduction pathways and reactive oxygen species (ROS) (Xu et al., 2019).

Interaction of quercetin with cell cycle regulatory proteins triggers a G2/M phase cell cycle arrest through the activation of the transcription factor p53, that has been suggested as a potential target for cancer therapy (Haupt et al., 2003). Quercetin is reported to induce a p53-p21 mediated cell cycle arrest at the G2/M phase and to suppress the NF-κB pathway, thus inhibiting the proliferation of HeLa cells (Priyadarsini et al., 2010). In another study, quercetin has been shown to induce apoptosis and antioxidant activity by two-fold in colon cancer cells (Atashpour et al., 2015).

Along with the antioxidant, anti-inflammatory and immunoprotective effects, quercetin has also been reported to show antimutagenic properties which may account for its role in chemoprevention. Shivakumar et al., have demonstrated the protective role of quercetin against a set of mutagens using a series of tests including Ames test, mice bone marrow micronucleus test, cell gene mutation test and chromosomal aberration test. According to this study Quercetin displays significant antimutagenicity against several mutagens such as sodium azide, benzo(a)pyrene, cyclophosphamide monohydrate, methyl methane sulphonate and etoposide (Shivakumar et al., 2017).

## Genistein

Genistein, (4′, 5, 7-trihydroxyisoflavone), an isoflavone with a heterocyclic diphenolic structure found in soy-based foods and legumes, has been extensively investigated to determine its chemopreventive and therapeutic activities (El-Rayes et al., 2011).

Several antitumor studies have shown that genistein inhibits the process of carcinogenesis through cell cycle regulation, induction of apoptosis, modulations in the signal transduction pathways and inhibition of angiogenesis.

Studies have demonstrated the chemoprotective effect of genistein against breast cancer, irrespective of the receptor status of the human breast cancer cell lines (Shon et al., 2006). COX-2 overexpression and increased CYP1A1 and ornithine decarboxylase (ODC) activity are frequently observed patterns in human breast cancer (Half et al., 2002; Deng et al., 2008; Androutsopoulos et al., 2009). Reports suggest that genistein inhibits the expression and activity of COX-2, CYP1A1 and ODC indicating the potential of genistein to be used as a chemopreventive.

A clear dose dependent antimutagenic effect of genistein has been reported against the mutagens, Aflatoxin B1 (AFB_1_), 3-methylimidazo (4, 5-f) quinoline (IQ) and N-nitroso-N-methyl urea (MNU) (Polivkova et al., 2006). Genistein also exhibited dose dependent inhibition of mutagenicity of PhIP 2-amino-1-methyl-6-phenylimidazol (4, 5-b) pyridine, a heterocyclic amine (Weisburger et al., 1998).

Another study has found that genistein induces apoptosis in colon cancer cells by up-regulating caspase-3 gene expression and inhibiting the proliferation and migration of the cancer cells The study also reports a down-regulation of p38 MAPK gene expression and a decrease in the level of p38 MAPK protein by genistein in colon cancer cells (Shafiee et al., 2016).

Anticancer efficacy of genistein has been demonstrated in preclinical models of gastric cancer. The study reveals that genistein mediates the down-regulation of the expression of the antiapoptotic protein B cell lymphoma 2 (Bcl-2) and up-regulation of the expression of proapoptotic Bcl-2 associated X protein (Bax) (Zhou et al., 2004). In another study, human gastric cancer cells (SGC7901) were injected subcutaneously in nude mice followed by direct administration of different doses of genistein at a site adjacent to the tumor. A decrease in tumor size was observed in all groups administered with genistein. The study revealed that genistein induces apoptosis by decreasing the Bcl-2/Bax ratio, suggesting its efficacy against preventing gastric carcinogenesis (Zhou et al., 2008). Another study has demonstrated the pro-apoptotic and antiproliferative effect of genistein against gastric carcinogenesis. Here genistein suppressed the NF-κB pathway, consequently reducing the levels of COX-2 (Li et al., 2011).

Genistein has been reported to exhibit its anti-inflammatory effect by inhibiting the expression of inflammatory cytokines (Jeong et al., 2014). More recently, the anti-inflammatory and anticancer effect of long-term genistein treatment was reported in diethyl nitrosamine-induced liver carcinogenesis model. A consistent increase in the levels of phospho-AMPK has been reported along with a down-regulation of the pro-inflammatory cytokines, TNF and IL-6. Genistein was also found to increase the level of p53, leading to the induction of apoptotic markers. Altogether, these results indicate that long-term dietary intake of genistein would aid in the prevention of hepatocellular carcinogenesis (Lee et al., 2019).

The inhibitory effect of genistein on NF-κB was further explored by molecular docking analysis, where the binding interaction of genistein with the active sites of NF-κB proteins was studied. The findings from the in silico analysis suggested that the amino acids (Lys52, Ser243, Asp274, Lys275) might play a pivotal role in anti- breast cancer activity (Mukund 2020).

Research findings also revealed an antimetastatic role of genistein in colon cancer cells and salivary adenoid cystic carcinoma cells. Genistein inhibited COX-2, MMP9, Ang-1, vasodilator-stimulated phosphoprotein and VEGF in HCT116 (Kang et al., 2018). Similarly, a decrease in the expression of VEGF and MMP-9 was observed in salivary adenoid cystic carcinoma, following treatment with genistein (Liu and Yu, 2004).

**FIGURE 4:**
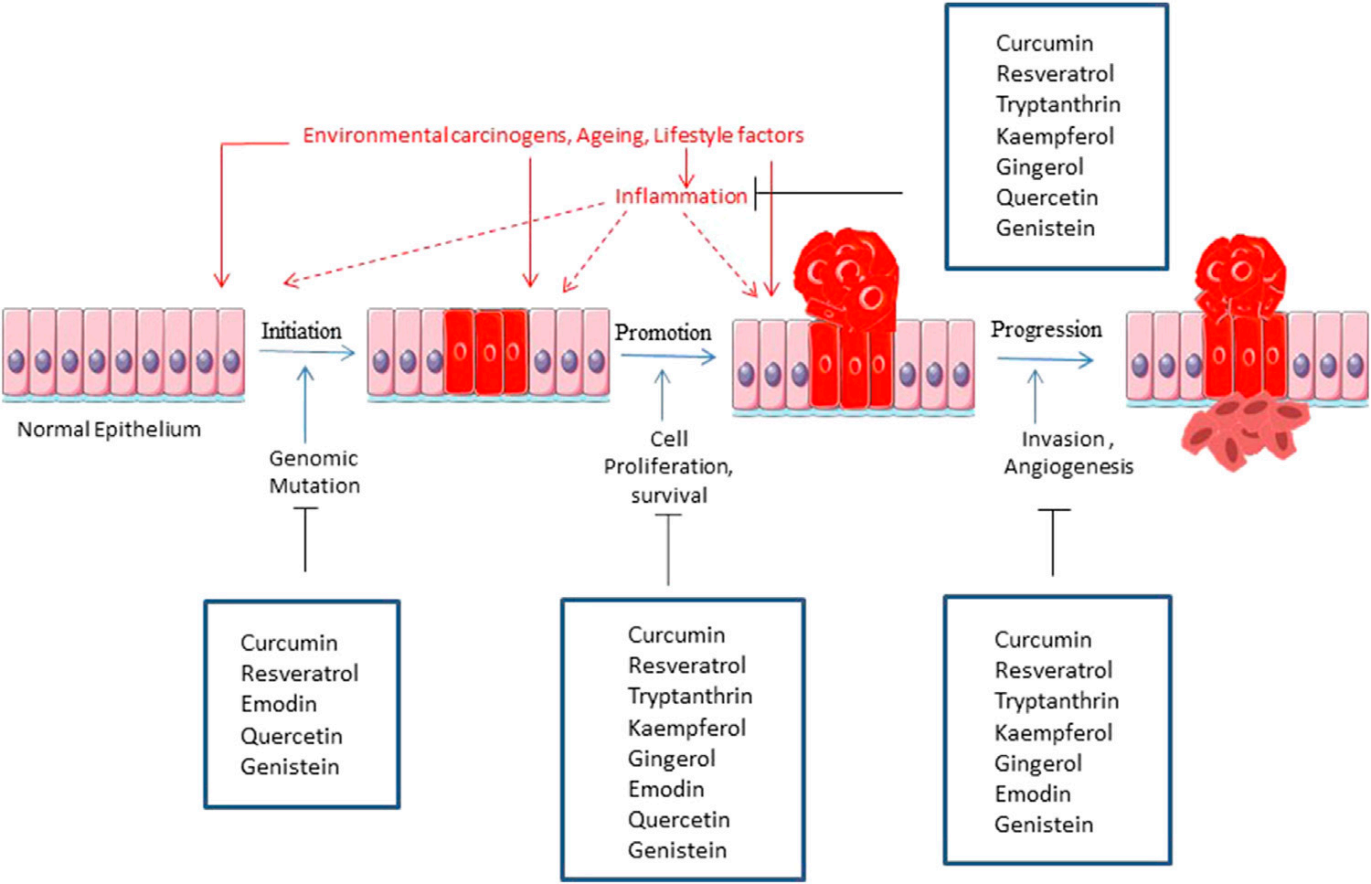
Illustration of various steps of carcinogenesis inhibited by the phytochemicals.

Figure 4 is a graphical representation of the inhibition of major tumorigenic factors by the phytochemicals.

**FIGURE 5:**
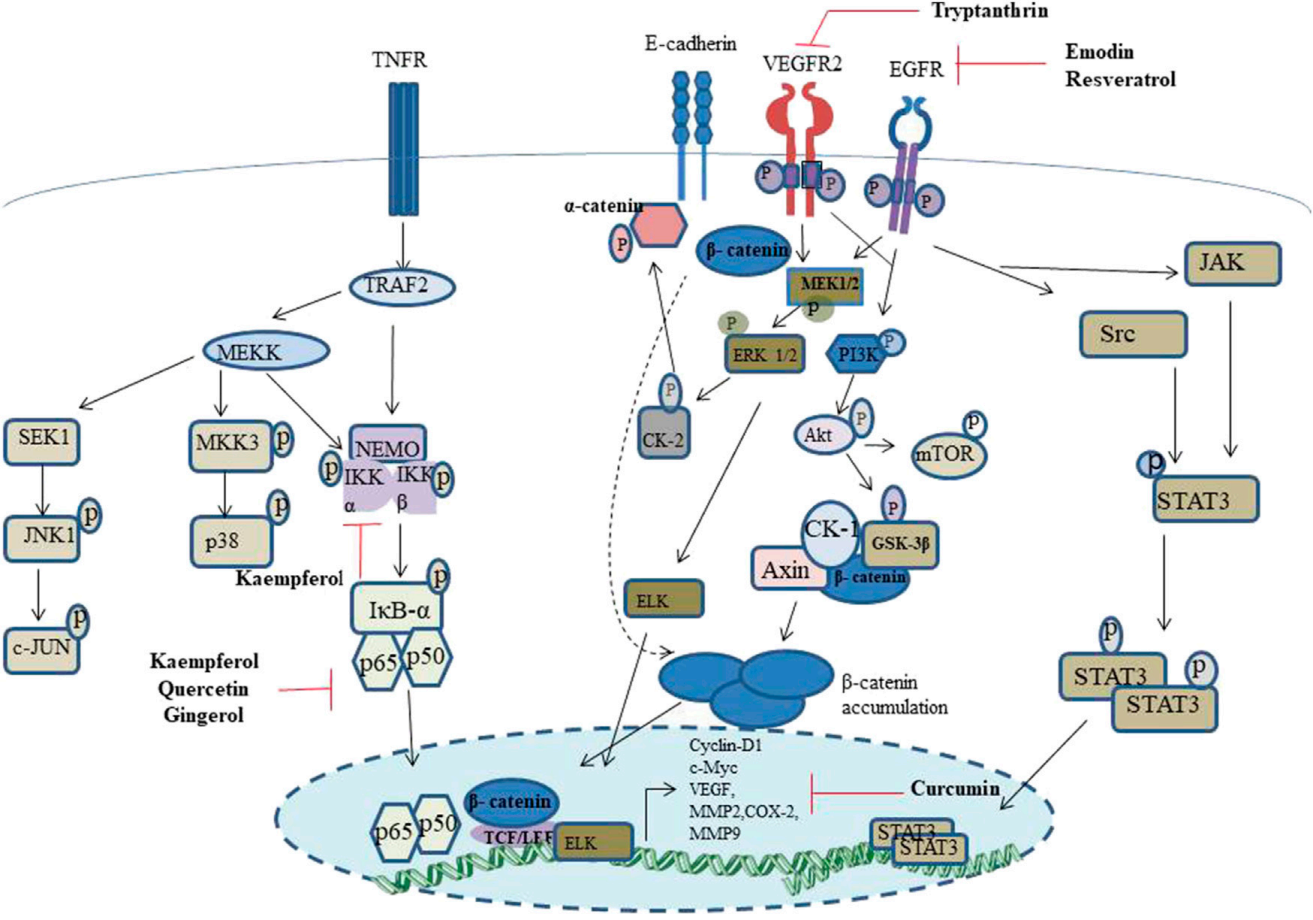
Schematic representation of the major signalling events regulated by the phytochemicals discussed in the current report.

Figure 5 is a graphical account of the major signalling events modulated by the chemopreventives discussed in this report.

Table 3 summarizes the important signaling pathways and key target molecules modulating the chemopreventive potential of phytochemicals.

**TABLE 3:** Signaling pathways modulated by the phytochemicals explored as chemopreventives and their molecular targets in different cancers.

Compound	Molecular targets	Cancer	Pathway	References
Tryptanthrin	VEGFR2 Liao et al. (2013)	Chronic myeloid leukemia	Bax, Bcl2, Cytochrome C, Caspase 3	Miao et al. (2011)
		Neuroblastoma	N-myc	Liao and Leung (2013)
		Breast cancer	MDR1, p53, P-glycoprotein, GST II, JNK	Yu et al. (2007), Yu et al. (2009)
		Monocytic and Promyelocytic leukemia	Caspase 3/FAS Antigen pathway	Kimoto et al. (2001)
		Epithelial colorectal adenocarcinoma	P-gp, MRP-2	Zhu et al. (2011)
		Skin cancer	MAPK, β-catenin	Shankar G et al. (2020)
Curcumin	M-CSF 1 receptor, aldo-keto reductase family 1 member C3, amiloride-sensitive amine oxidase and tyrosine-protein phosphatase non-receptor type 11 MMP-2, NAD-dependent protein deacetylase sirtuin-2, core histone macro-H2A.1, NAD-dependent protein deacetylase sirtuin-1 and epidermal growth factor receptor Furlan et al. (2018)	Myeloid leukemia	Cytochrome C-PARP-Caspase 9 cleavage	Mutlu Altundağ et al. (2021)
		Melanoma	ROS-GSH-MMP	Liao et al. (2017)
		Gastric cancer	ROS-ASK1-JNK	Liang et al. (2014)
		Breast cancer	Cyclin B1, CDC2, NF-κB	Akkoç et al. (2016)
	Phosphodiesterase 4 (PDE4) Furlan and Bren (2021)	Glioblastoma	MAPK, NF-κB, JAK/STAT3-IAP	Gersey et al. (2017)
		Liver cancer	MAPK, NF-κB, JAK/STAT3-IAP	Marquardt et al. (2015)
Kaempferol	NF-κBIKK Kadioglu et al. (2015)	Breast cancer	MAPK-ERK-MEK1&ELK1	Kim et al. (2008)
			AP-1-Cathepsin B&D, MMP-2, MMP-9	
		Brain cancer	Bcl2-Cleaved caspase 3,8, XIAP, Cleaved PARP	Sharma et al. (2007)
		Liver cancer	STAT3-CDK1, Cyclin B, PI3K/AKT/mTOR	Huang et al. (2013)
		Gastric cancer	IRE1-JNK-CHOP	Kim et al. (2018)
		Lung cancer	AKT/PI3K&ERK PTEN, Bax, miR-340, Fas, cleaved caspase3,8,9 and cleaved PARP	Nguyen et al. (2003), Han et al. (2018)
		Pancreatic cancer	EGFR, AKT, Src, ERK1	Lee and Kim (2016)
		Stomach cancer	COX2, Bcl2, p-ERK,p-AKT, Bax, Cleaved caspase 3,9	Song et al. (2015)
		Oral cancer	MMP2, TIMP2, C-jun, ERK1/2	Lin et al. (2013)
Resveratrol	LSD1 NTMT1 BIRC4 Kores et al. (2019)	Breast cancer	p53, AKT, NF-κB	Athar et al. (2009)
	EGF Kumara et al. (2017)	Prostate cancer	PI3K/AKT, AMPK	Rashid et al. (2011)
		Colon cancer	AMPK, p53	Liu et al. (2019)
		Pancreatic cancer	NF-κB	Mouria et al. (2002)
		Ovarian cancer	ERK, NF-κB	Tino et al. (2016)
		Epidermoid carcinoma	MEK-1, AP-1	Kim et al. (2006)
		Osteosarcoma	pERK1/2	Alkhalaf and Jaffal (2006)
		Squamous cell carcinoma	MEK, VEGF, AKT	Athar et al. (2009)
		Leukemia	mTOR and p38 MAPK	Jiao et al. (2013)
		Lung cancer	mTOR and p38 MAPK	Wang et al. (2018)
Gingerol	PI3K NF-κB C-Met COX2 Kumara et al. (2017)	Cervical cancer	PI3K/AKT AMPK mTOR	Zhang et al. (2021)
	LTA_4_H (El-Naggar et al., 2017)	Skin cancer	COX-2, NF-κB and p38 MAPK	Kim et al. (2005)
		Breast cancer	PI3K/AKT and p38 MAPK	Joo et al. (2016)
		Colon cancer	LTA_4_H	Jeong et al. (2009)
Emodin	FGFR2 Chen et al. (2013)	Colorectal cancer	VEGF, Wnt	Dai et al. (2019), Gu et al. (2019)
		Breast cancer	ERα-MAPK, AKT-Cyclin D1/Bcl-2 Her2	Zhang et al. (1995), Sui et al. (2014)
		Cervical cancer	PI3K/AKT, TGF-β	Olsen et al. (2007)
		Lung cancer	PKC	Lee (2001)
		Pancreatic cancer	EGFR, STAT3 NF-κB	Wang et al. (2019), Tong et al. (2020)
		Head and neck squamous cell carcinoma	β-catenin, AKT	Way et al. (2014)
		Hepatocellular carcinoma	STAT3, PI3K/AKT/mTOR	Subramaniam et al. (2013)
Quercetin	NF-κB Mukund et al. (2019)	Prostate cancer	PI3/AKT Ras/Raf/MEK/MAPK	Sharmila et al. (2014)
		Oral squamous cell carcinoma	NF-κB	Sun et al. (2010)
		Gastric cancer	PI3/AKT	Shen et al. (2016)
		Brain cancer	JAK 2/STAT 3	Wang et al. (2013)
		Skin cancer	MEK, ERK, PI3/AKT	Rafiq et al. (2015)
		Mesothelioma cancer	JNK, p38, MAPK/ERK	Demiroglu-Zergeroglu et al. (2016)
Genistein	ERα Pang et al. (2018)	Breast cancer	(a) NF-κB (b) Notch 1-NF-κB	Pan et al. (2012), Mukund (2020)
		Prostate cancer	PI3/AKT	Li and Sarkar (2002)
		Colon Cancer	(a) Notch 1/NF-κB Slug/E-cadherin (b)Wnt pathway	Zhang and Chen (2011), Zhou et al. (2017)
		Endometrial cancer	AKT/mTOR, MAPK	Malloy et al. (2018)
		Esophageal cancer	JAK1/2, STAT3 and AKT/MDM2/p53	Gao et al. (2020)

Table 4 provides a brief description of the important studies conducted for improving the pharmacokinetics of the chemoprevention strategies of the prospective phytochemicals.

**TABLE 4:** Strategic approaches aimed at improving the pharmacokinetics of prospective chemopreventives.

Compound	Approach	References
Curcumin	Chitosan encapsulation	Vijayakurup et al. (2019)
	PLGA encapsulation	Nair et al. (2012)
Resveratrol	Co-administration with piperine	Johnson et al. (2011)
	Combination with Magnesium dihydroxide based formulation	—
Kaempferol	Poly (ethyleneoxide)-poly (propyleneoxide)-poly (ethylene oxide) encapsulation	Luo et al. (2012)
	PLGA encapsulation	Luo et al. (2012)
Emodin	Co-administration with piperine	Di et al. (2015)

## Challenges and Future Directions

The idea of chemoprevention, especially primary chemoprevention, requires identification of a population susceptible to a particular type of cancer. Depending on genetic, epigenetic, dietary habits and medical history, individuals vary in their susceptibility towards developing cancer when exposed to a carcinogen. Hence, a more accurate identification of susceptible individuals based on these parameters is essential for initiating a chemoprevention intervention. In addition, narrowing down the timeframe to estimate the time point at which the intervention has to be commenced presents a significant impediment to the whole idea. Moreover, the appropriate dose of the chemopreventive agent must also be estimated. This is important as inappropriate usage might result in highly deleterious side effects. For example, chronic use or high doses of aspirin may result in gastric haemorrhages (Harker et al., 1999); other chemopreventives like tamoxifen also has side effects (Yang et al., 2013). Considering this, discovery of biomarkers to more accurately identify and stratify individuals according to risk of cancer incidence might prove to be advantageous while designing a chemoprevention regimen.

Another challenge in cancer chemoprevention is to identify the individuals who might show a positive/negative outcome following a chemoprevention intervention. For example, a study was conducted to assess the influence of genotypic variation of *NKX3.1* on prostate cancer chemoprevention in the SELECT trial. The study assessed the influence of two prostate cancer-related polymorphisms, rs11781886 and rs2228013, in the *NKX3.1* on prostate cancer incidence following selenium or vitamin E administration. Their results showed a significant influence of NKX3.1 genotypes on increased tumor incidence on subjects who took selenium or vitamin E (Martinez et al., 2014). Likewise, another study identified that common single-nucleotide polymorphisms (SNP) in proximity or in the *ZNF423* or *CTSO* genes are associated with the effectiveness of chemoprevention approaches using selective estrogen receptor modulators (SERM). Mechanistic studies showed that these genes are associated with *BRCA1* expression in a SNP-dependent manner (Ingle et al., 2013). Some studies show that vitamin intake by subjects with vitamin deficiencies leads to positive chemopreventive effects while intake by those without deficiency did not show a positive outcome.

Discovery of candidates for chemoprevention trials in the clinic is based on epidemiological observations or data from preclinical studies. However, clinical trials based on epidemiological clues have often resulted in increased tumorigenesis. This could be due to the fact that molecules often act in multiple combinations to exert a protective effect, and hence a similar effect may not be observed with single molecules. Rather than relying on epidemiological data to design clinical trials, the mechanisms that drive the progression of these tumors and the anti-cancer activity of the compounds must be studied. A caveat in cancer drug discovery is the testing of the compound of interest in an appropriate model. Choosing a model that bears appreciable similarity with human tumors is critical as tumorigenesis is a multifactorial process and hence, individual factors that may be crucial for the outcome of the result may not be easily predictable. An example for this is the unexpected observation from a preclinical study on the effectiveness of finasteride against prostate cancer. Here, while the compound did not have effect on the growth of LNCaP cells, it increased the growth of tumors that developed from a combination of LNCaP cells and fibroblasts. Considering the significance of fibroblasts in prostate cancer, combination of fibroblasts and cancer cells is a better model for prostate cancer growth than a tumor originating from cancer cells alone. Interestingly, finasteride administration increased the incidence of high grade tumors in clinical trial.

## Conclusion

Cancer chemoprevention is a good strategy to mitigate the morbidity/mortality associated with tumor incidence. The success of chemoprevention in bringing down the mortality associated with cardiovascular disease has further reinforced the idea of implementing this strategy in lowering cancer incidence and associated mortality. Identification of major risk factors such as inherited mutations and exposure to environmental carcinogens is essential for designing appropriate chemoprevention intervention. Multiple clinical trials have shown success in lowering tumor incidence in susceptible population; however, multiple factors like the identification of susceptible individuals, difficulty in fixing time frame for chemoprevention intervention, and risk factors associated with chemoprevention could deter the adoption of this strategy on a larger scale. For example, less than 10% of women in the high-risk group who are offered an anti-estrogen medication as a primary chemopreventive, agree to take it (Crew et al., 2017). Discovery of novel, safe and effective chemopreventives is essential for this idea to gain more acceptability. The use of appropriate preclinical models that, to certain extend, mimics human tumorigenesis could lead to more success of candidate compounds in clinical trials. This is important considering the duration of clinical trials involved in discovering cancer chemopreventives. Moreover, clinical trials for primary chemopreventives are done on an at-risk population which doesn’t have tumor incidence at the time of the trial. Hence, clinical trial design must be based on sufficient experimental data regarding its safety, efficacy and mechanism of action so as to avert or minimize incidences of increased tumor burden in the treatment group.

## References

[B1] Abusnina A. Keravis T. Zhou Q. Justiniano H. Lobstein A. Lugnier C. (2015). Tumour Growth Inhibition and Anti-angiogenic Effects Using Curcumin Correspond to Combined PDE2 and PDE4 Inhibition. Thromb. Haemost. 113 (02), 319–328. 10.1160/TH14-05-0454 25230992

[B2] Ak T. Gülçin I. (2008). Antioxidant and Radical Scavenging Properties of Curcumin. Chem. Biol. Interact 174 (1), 27–37. 10.1016/j.cbi.2008.05.003 18547552

[B3] Akhtar S. Meeran S. M. Katiyar N. Katiyar S. K. (2009). Grape Seed Proanthocyanidins Inhibit the Growth of Human Non-small Cell Lung Cancer Xenografts by Targeting Insulin-like Growth Factor Binding Protein-3, Tumor Cell Proliferation, and Angiogenic Factors. Clin. Cancer Res. 15 (3), 821–831. 10.1158/1078-0432.CCR-08-1901 19188152

[B4] Akkol E. K. Tatlı I. I. Karatoprak G. Ş. Ağar O. T. Yücel Ç. Sobarzo-Sánchez E. (2021). Is Emodin with Anticancer Effects Completely Innocent? Two Sides of the Coin. Cancers (Basel) 13 (11), 2733. 10.3390/cancers13112733 34073059 PMC8198870

[B5] Akkoç Y. Arısan E. D. Çoker Gürkan A. Obakan Yerlikaya P. Palavan Ünsal Z. N. Berrak Ö. (2016). "The Inhibition of PI3K and NF Kappa B Promoted Curcumin-Induced Cell Cycle Arrest at G2/M via Altering Polyamine Metabolism in Bcl-2 Overexpressing MCF-7 Breast Cancer Cells." 10.1016/j.biopha.2015.12.007 26796279

[B6] Albanes D. Heinonen O. P. Huttunen J. K. Taylor P. R. Virtamo J. Edwards B. K. (1995). Effects of Alpha-Tocopherol and Beta-Carotene Supplements on Cancer Incidence in the Alpha-Tocopherol Beta-Carotene Cancer Prevention Study. Am. J. Clin. Nutr. 62 (6), 1427S–1430S. 10.1093/ajcn/62.6.1427S 7495243

[B7] Alkhalaf M. Jaffal S. (2006). Potent Antiproliferative Effects of Resveratrol on Human Osteosarcoma SJSA1 Cells: Novel Cellular Mechanisms Involving the ERKs/p53 cascade. Free Radic. Biol. Med. 41 (2), 318–325. 10.1016/j.freeradbiomed.2006.04.019 16814113

[B8] Allegra A. Innao V. Russo S. Gerace D. Alonci A. Musolino C. (2017). Anticancer Activity of Curcumin and its Analogues: Preclinical and Clinical Studies. Cancer Invest. 35 (1), 1–22. 10.1080/07357907.2016.1247166 27996308

[B9] Amin A. R. M. R. Wang D. Nannapaneni S. Lamichhane R. Chen Z. G. Shin D. M. (2021). Combination of Resveratrol and green tea Epigallocatechin Gallate Induces Synergistic Apoptosis and Inhibits Tumor Growth *In Vivo* in Head and Neck Cancer Models. Oncol. Rep. 45 (5), 1–10. 10.3892/or.2021.8038 33864659 PMC8025073

[B10] Andriole G. L. Bostwick D. G. Brawley O. W. Gomella L. G. Marberger M. Montorsi F. (2010). Effect of Dutasteride on the Risk of Prostate Cancer. N. Engl. J. Med. 362, 1192–1202. 10.1056/NEJMoa0908127 20357281

[B11] Androutsopoulos V. P. Tsatsakis A. M. Spandidos D. A. (2009). Cytochrome P450 CYP1A1: Wider Roles in Cancer Progression and Prevention. BMC cancer 9 (1), 1–17. 10.1186/1471-2407-9-187 19531241 PMC2703651

[B12] Anto R. J. Mukhopadhyay A. Denning K. Aggarwal B. B. (2002). Curcumin (Diferuloylmethane) Induces Apoptosis through Activation of Caspase-8, BID Cleavage and Cytochrome C Release: its Suppression by Ectopic Expression of Bcl-2 and Bcl-Xl. Carcinogenesis 23 (1), 143–150. 10.1093/carcin/23.1.143 11756235

[B13] Atashpour S. Fouladdel S. Movahhed T. K. Barzegar E. Ghahremani M. H. Ostad S. N. (2015). Quercetin Induces Cell Cycle Arrest and Apoptosis in CD133+ Cancer Stem Cells of Human Colorectal HT29 Cancer Cell Line and Enhances Anticancer Effects of Doxorubicin. Iranian J. Basic Med. Sci. 18 (7), 635. PMC4556754 26351552

[B14] Athar M. Back J. H. Kopelovich L. Bickers D. R. Kim A. L. (2009). Multiple Molecular Targets of Resveratrol: Anti-carcinogenic Mechanisms. Arch. Biochem. Biophys. 486 (2), 95–102. 10.1016/j.abb.2009.01.018 19514131 PMC2749321

[B15] Azevedo C. Correia-Branco A. Araújo J. R. Guimaraes J. T. Keating E. Martel F. (2015). The Chemopreventive Effect of the Dietary Compound Kaempferol on the MCF-7 Human Breast Cancer Cell Line Is Dependent on Inhibition of Glucose Cellular Uptake. Nutr. Cancer 67 (3), 504–513. 10.1080/01635581.2015.1002625 25719685

[B16] Bava S. V. Sreekanth C. N. Thulasidasan A. K. T. Anto N. P. Cheriyan V. T. Puliyappadamba V. T. (2011). Akt Is Upstream and MAPKs Are Downstream of NF-Κb in Paclitaxel-Induced Survival Signaling Events, Which Are Down-Regulated by Curcumin Contributing to Their Synergism. Int. J. Biochem. Cel. Biol. 43 (3), 331–341. 10.1016/j.biocel.2010.09.011 20883815

[B17] Bhat K. P. Pezzuto J. M. (2002). Cancer Chemopreventive Activity of Resveratrol. Ann. N Y Acad. Sci. 957 (1), 210–229. 10.1111/j.1749-6632.2002.tb02918.x 12074974

[B18] Boadi W. Y. Myles E. L. Garcia A. S. (2020). Phospho Tensin Homolog in Human and Lipid Peroxides in Peripheral Blood Mononuclear Cells Following Exposure to Flavonoids. J. Am. Coll. Nutr. 39 (2), 135–146. 10.1080/07315724.2019.1616234 31192773 PMC6908767

[B19] Bobe G. Albert P. S. Sansbury L. B. Lanza E. Schatzkin A. Colburn N. H. (2010). Interleukin-6 as a Potential Indicator for Prevention of High-Risk Adenoma Recurrence by Dietary Flavonols in the Polyp Prevention Trial. Cancer Prev. Res. 3 (6), 764–775. 10.1158/1940-6207.CAPR-09-0161 PMC2881177 20484173

[B20] Boldry E. J. Yuan J.-M. Carmella S. G. Wang R. Tessier K. Hatsukami D. K. (2020). Effects of 2-phenethyl Isothiocyanate on Metabolism of 1, 3-butadiene in Smokers. Cancer Prev. Res. 13 (1), 91–100. 10.1158/1940-6207.CAPR-19-0296 PMC8166320 31771940

[B21] Bourguignon L. Y. Xia W. Wong G. (2009). Hyaluronan-mediated CD44 Interaction with P300 and SIRT1 Regulates β-catenin Signaling and NFκB-specific Transcription Activity Leading to MDR1 and Bcl-xL Gene Expression and Chemoresistance in Breast Tumor Cells. J. Biol. Chem. 284 (5), 2657–2671. 10.1074/jbc.M806708200 19047049 PMC2631959

[B22] Brasky T. M. Baik C. S. Slatore C. G. Potter J. D. White E. (2012). Non-steroidal Anti-inflammatory Drugs and Small Cell Lung Cancer Risk in the VITAL Study. Lung cancer 77 (2), 260–264. 10.1016/j.lungcan.2012.04.015 22608142 PMC3552491

[B23] Brglez Mojzer E. Knez Hrnčič M. Škerget M. Knez Ž. Bren U. (2016). Polyphenols: Extraction Methods, Antioxidative Action, Bioavailability and Anticarcinogenic Effects. Molecules 21 (7), 901. 10.3390/molecules21070901 PMC6273793 27409600

[B24] Buhrmann C. Shayan P. Popper B. Goel A. Shakibaei M. (2016). Sirt1 Is Required for Resveratrol-Mediated Chemopreventive Effects in Colorectal Cancer Cells. Nutrients 8 (3), 145. 10.3390/nu8030145 26959057 PMC4808874

[B25] Cai T. Di Vico T. Durante J. Tognarelli A. Bartoletti R. (2018). Human Papilloma Virus and Genitourinary Cancers: a Narrative Review. Minerva Urologica e Nefrologica= Ital. J. Urol. Nephrol. 70 (6), 579–587. 10.23736/S0393-2249.18.03141-7 30160386

[B26] Cancer C. G. o. H. F. i. B. (2002). Alcohol, Tobacco and Breast Cancer–Collaborative Reanalysis of Individual Data from 53 Epidemiological Studies, Including 58 515 Women with Breast Cancer and 95 067 Women without the Disease. Br. J. Cancer 87 (11), 1234. 12439712 10.1038/sj.bjc.6600596 PMC2562507

[B27] Cha T.-L. Qiu L. Chen C.-T. Wen Y. Hung M.-C. (2005). Emodin Down-Regulates Androgen Receptor and Inhibits Prostate Cancer Cell Growth. Cancer Res. 65 (6), 2287–2295. 10.1158/0008-5472.CAN-04-3250 15781642

[B28] Chapman A. M. Sun K. Y. Ruestow P. Cowan D. M. Madl A. K. (2016). Lung Cancer Mutation Profile of EGFR, ALK, and KRAS: Meta-Analysis and Comparison of Never and Ever Smokers. Lung Cancer 102, 122–134. 10.1016/j.lungcan.2016.10.010 27987580

[B29] Chen A. C. Martin A. J. Choy B. Fernández-Peñas P. Dalziell R. A. McKenzie C. A. (2015). A Phase 3 Randomized Trial of Nicotinamide for Skin-Cancer Chemoprevention. New Engl. J. Med. 373 (17), 1618–1626. 10.1056/nejmoa1506197 26488693

[B30] Chen A. Y. Chen Y. C. (2013). Multipotent Flavonoid Kaempferol: Molecular Targets and Mechanism of Action and Nanotechnology Applications in Cancer and Human Health. Chem. Phys. Res. J. 6 (3/4), 379.

[B31] Chen C. Kong A.-N. T. (2005). Dietary Cancer-Chemopreventive Compounds: from Signaling and Gene Expression to Pharmacological Effects. Trends Pharmacological Sciences 26 (6), 318–326. 10.1016/j.tips.2005.04.004 15925707

[B32] Chen G. Qiu H. Ke S.-D. Hu S.-M. Yu S.-Y. Zou S.-Q. (2013). Emodin Regulating Excision Repair Cross-Complementation Group 1 through Fibroblast Growth Factor Receptor 2 Signaling. World J. Gastroenterol. WJG 19 (16), 2481. 10.3748/wjg.v19.i16.2481 23674849 PMC3646138

[B33] Cox M. E. Gleave M. E. Zakikhani M. Bell R. H. Piura E. Vickers E. (2009). Insulin Receptor Expression by Human Prostate Cancers. The Prostate 69 (1), 33–40. 10.1002/pros.20852 18785179

[B34] Crew K. D. Albain K. S. Hershman D. L. Unger J. M. Lo S. S. (2017). How Do We Increase Uptake of Tamoxifen and Other Anti-estrogens for Breast Cancer Prevention? NPJ Breast Cancer 3 (1), 1–7. 10.1038/s41523-017-0021-y 28649660 PMC5460136

[B35] Cuzick J. Forbes J. F. Sestak I. Cawthorn S. Hamed H. Holli K. (2007). Long-term Results of Tamoxifen Prophylaxis for Breast Cancer—96-Month Follow-Up of the Randomized IBIS-I Trial. J. Natl. Cancer Inst. 99 (4), 272–282. 10.1093/jnci/djk049 17312304

[B36] Cuzick J. Sestak I. Bonanni B. Costantino J. P. Cummings S. DeCensi A. (2013). Selective Oestrogen Receptor Modulators in Prevention of Breast Cancer: an Updated Meta-Analysis of Individual Participant Data. The Lancet 381 (9880), 1827–1834. 10.1016/S0140-6736(13)60140-3 PMC3671272 23639488

[B37] Dai G. Ding K. Cao Q. Xu T. He F. Liu S. (2019). Emodin Suppresses Growth and Invasion of Colorectal Cancer Cells by Inhibiting VEGFR2. Eur. J. Pharmacol. 859, 172525. 10.1016/j.ejphar.2019.172525 31288005

[B38] Danz H. Baumann D. Hamburger M. (2002). Quantitative Determination of the Dual COX-2/5-LOX Inhibitor Tryptanthrin in Isatis Tinctoria by ESI-LC-MS. Planta Med. 68 (02), 152–157. 10.1055/s-2002-20252 11859467

[B39] Danz H. Stoyanova S. Thomet O. A. Simon H.-U. Dannhardt G. Ulbrich H. (2002). Inhibitory Activity of Tryptanthrin on Prostaglandin and Leukotriene Synthesis. Planta Med. 68 (10), 875–880. 10.1055/s-2002-34922 12391548

[B40] Danz H. Stoyanova S. Wippich P. Brattström A. Hamburger M. (2001). Identification and Isolation of the Cyclooxygenase-2 Inhibitory Principle in Isatis Tinctoria. Planta Med. 67 (05), 411–416. 10.1055/s-2001-15805 11488453

[B41] Demiroglu-Zergeroglu A. Ergene E. Ayvali N. Kuete V. Sivas H. (2016). Quercetin and Cisplatin Combined Treatment Altered Cell Cycle and Mitogen Activated Protein Kinase Expressions in Malignant Mesotelioma Cells. BMC Complement. Altern. Med. 16 (1), 1–6. 10.1186/s12906-016-1267-x 27514524 PMC4982421

[B42] Deng W. Jiang X. Mei Y. Sun J. Ma R. Liu X. (2008). Role of Ornithine Decarboxylase in Breast Cancer. Acta Biochim. Biophys. Sinica 40 (3), 235–243. 10.1111/j.1745-7270.2008.00397.x 18330478

[B43] Dhillon N. Aggarwal B. B. Newman R. A. Wolff R. A. Kunnumakkara A. B. Abbruzzese J. L. (2008). Phase II Trial of Curcumin in Patients with Advanced Pancreatic Cancer. Clin. Cancer Res. 14 (14), 4491–4499. 10.1158/1078-0432.CCR-08-0024 18628464

[B44] Di X. Wang X. Liu Y. (2015). Effect of Piperine on the Bioavailability and Pharmacokinetics of Emodin in Rats. J. Pharm. Biomed. Anal. 115, 144–149. 10.1016/j.jpba.2015.06.027 26201645

[B45] Divya C. S. Pillai M. R. (2006). Antitumor Action of Curcumin in Human Papillomavirus Associated Cells Involves Downregulation of Viral Oncogenes, Prevention of NFkB and AP‐1 Translocation, and Modulation of Apoptosis. Mol. Carcinogenesis: Published cooperation Univ. Tex. MD Anderson Cancer Cent. 45 (5), 320–332. 10.1002/mc.20170 16526022

[B46] Dong X. Fu J. Yin X. Cao S. Li X. Lin L. Huyiligeqi Y. (2016). Emodin: A review of its pharmacology, toxicity and pharmacokinetics. Phytotherapy Research 30 (8), 1207–1218. 10.1002/ptr.5631 27188216 PMC7168079

[B47] El-Rayes B. F. Philip P. A. Sarkar F. H. Shields A. F. Ferris A. M. Hess K. Kaseb A. O. Javle M. M. Varadhachary G. R. Wolff R. A. (2011). A phase II study of isoflavones, erlotinib, and gemcitabine in advanced pancreatic cancer. Investigational new drugs 29 (4), 694–699. 10.1007/s10637-010-9386-6 20107864

[B48] Elmets C. A. Viner J. L. Pentland A. P. Cantrell W. Lin H.-Y. Bailey H. Kang S. Linden K. G. Heffernan M. Duvic M. (2010). Chemoprevention of nonmelanoma skin cancer with celecoxib: a randomized, double-blind, placebo-controlled trial. Journal of the National Cancer Institute 102 (24), 1835–1844. 10.1093/jnci/djq442 21115882 PMC3001966

[B49] Esmat A. Y. Refaie F. M. Shaheen M. H. Said M. M. (2002). Chemoprevention of prostate carcinogenesis by DFMO and/or finasteride treatment in male Wistar rats. Tumori Journal 88 (6), 513–521. 10.1177/030089160208800616 12597149

[B50] Fang Y.-P. Lin Y.-K. Su Y.-H. Fang J.-Y. (2011). Tryptanthrin-loaded nanoparticles for delivery into cultured human breast cancer cells, MCF7: the effects of solid lipid/liquid lipid ratios in the inner core. Chemical and Pharmaceutical Bulletin 59 (2), 266–271. 10.1248/cpb.59.266 21297310

[B51] Ferraz da Costa D. C. Pereira Rangel L. Quarti J. Santos R. A. Silva J. L. Fialho E. (2020). Bioactive Compounds and Metabolites from Grapes and Red Wine in Breast Cancer Chemoprevention and Therapy. Molecules 25 (15), 3531. 10.3390/molecules25153531 PMC7436232 32752302

[B52] Fiedler M. A. Wernke-Dollries K. Stark J. M. (1998). Inhibition of TNF-α-Induced NF-κ B Activation and IL-8 Release in A549 Cells with the Proteasome Inhibitor MG-132. Am. J. Respir. Cel Mol. Biol. 19 (2), 259–268. 10.1165/ajrcmb.19.2.3149 9698598

[B53] Fischer S. M. Lo H. H. Gordon G. B. Seibert K. Kelloff G. Lubet R. A. (1999). Chemopreventive Activity of Celecoxib, a Specific Cyclooxygenase‐2 Inhibitor, and Indomethacin against Ultraviolet Light–Induced Skin Carcinogenesis. Mol. carcinogenesis 25 (4), 231–240. 10.1002/(sici)1098-2744(199908)25:4<231:aid-mc1>3.0.co;2-f 10449029

[B54] Fisher B. Costantino J. P. Wickerham D. L. Redmond C. K. Kavanah M. Cronin W. M. (1998). Tamoxifen for Prevention of Breast Cancer: Report of the National Surgical Adjuvant Breast and Bowel Project P-1 Study. JNCI: J. Natl. Cancer Inst. 90 (18), 1371–1388. 10.1093/jnci/90.18.1371 9747868

[B55] Foote J. A. Ranger-Moore J. R. Einspahr J. G. Saboda K. Kenyon J. Warneke J. (2009). Chemoprevention of Human Actinic Keratoses by Topical Dl-α-Tocopherol. Cancer Prev. Res. 2 (4), 394–400. 10.1158/1940-6207.CAPR-08-0210 PMC4154592 19336724

[B56] Fu H. Zhang J. Pan J. Zhang Q. Lu Y. Wen W. (2011). Chemoprevention of Lung Carcinogenesis by the Combination of Aerosolized Budesonide and Oral Pioglitazone in A/J Mice. Mol. carcinogenesis 50 (12), 913–921. 10.1002/mc.20751 PMC6015734 21374736

[B57] Furlan V. Bren U. (2021). Insight into Inhibitory Mechanism of PDE4D by Dietary Polyphenols Using Molecular Dynamics Simulations and Free Energy Calculations. Biomolecules 11 (3), 479. 10.3390/biom11030479 33806914 PMC8004924

[B58] Furlan V. Bren U. (2020). Protective Effects of [6]-gingerol against Chemical Carcinogens: Mechanistic Insights. Int. J. Mol. Sci. 21 (3), 695. 10.3390/ijms21030695 PMC7037038 31973096

[B59] Furlan V. Konc J. Bren U. (2018). Inverse Molecular Docking as a Novel Approach to Study Anticarcinogenic and Anti-neuroinflammatory Effects of Curcumin. Molecules 23 (12), 3351. 10.3390/molecules23123351 PMC6321024 30567342

[B60] Gao J. Xia R. Chen J. Luo X. Ke C. Ren C. (2020). Inhibition of Esophageal-Carcinoma Cell Proliferation by Genistein via Suppression of JAK1/2-STAT3 and AKT/MDM2/p53 Signaling Pathways. Aging (Albany NY) 12 (7), 6240. 10.18632/aging.103019 32276266 PMC7185096

[B61] Gersey Z. C. Rodriguez G. A. Barbarite E. Sanchez A. Walters W. M. Ohaeto K. C. (2017). Curcumin Decreases Malignant Characteristics of Glioblastoma Stem Cells via Induction of Reactive Oxygen Species. BMC cancer 17 (1), 1–11. 10.1186/s12885-017-3058-2 28160777 PMC5292151

[B62] Goss P. E. Ingle J. N. Alés-Martínez J. E. Cheung A. M. Chlebowski R. T. Wactawski-Wende J. (2011). Exemestane for Breast-Cancer Prevention in Postmenopausal Women. New Engl. J. Med. 364 (25), 2381–2391. 10.1056/NEJMoa1103507 21639806

[B63] Group A.-T. B. C. C. P. S. (1994). The Effect of Vitamin E and Beta Carotene on the Incidence of Lung Cancer and Other Cancers in Male Smokers. New Engl. J. Med. 330 (15), 1029–1035. 10.1056/NEJM199404143301501 8127329

[B64] Gu J. Cui C.-f. Yang L. Wang L. Jiang X.-h. (2019). Emodin Inhibits colon Cancer Cell Invasion and Migration by Suppressing Epithelial–Mesenchymal Transition via the Wnt/β-Catenin Pathway. Oncol. Res. 27 (2), 193. 10.3727/096504018X15150662230295 29301594 PMC7848449

[B65] Guo H. Liu F. Yang S. Xue T. (2020). Emodin Alleviates Gemcitabine Resistance in Pancreatic Cancer by Inhibiting MDR1/P-Glycoprotein and MRPs Expression. Oncol. Lett. 20 (5), 1. 10.3892/ol.2020.12030 32934734 PMC7471752

[B66] Half E. Tang X. M. Gwyn K. Sahin A. Wathen K. Sinicrope F. A. (2002). Cyclooxygenase-2 Expression in Human Breast Cancers and Adjacent Ductal Carcinoma *In Situ* . Cancer Res. 62 (6), 1676–1681. 11912139

[B67] Han X. Liu C.-F. Gao N. Zhao J. Xu J. (2018). Kaempferol Suppresses Proliferation but Increases Apoptosis and Autophagy by Up-Regulating microRNA-340 in Human Lung Cancer Cells. Biomed. Pharmacother. 108, 809–816. 10.1016/j.biopha.2018.09.087 30253373

[B68] Haritha N. H. Nawab A. Vijayakurup V. Anto N. P. Liju V. B. Alex V. V. (2021). Targeting Thymidylate Synthase Enhances the Chemosensitivity of Triple-Negative Breast Cancer towards 5-FU-Based Combinatorial Therapy. Front. Oncol., 2741. 10.3389/fonc.2021.656804 PMC8320437 34336653

[B69] Harker L. A. Boissel J.-P. Pilgrim A. J. Gent M. (1999). Comparative Safety and Tolerability of Clopidogrel and Aspirin. Drug Saf. 21 (4), 325–335. 10.2165/00002018-199921040-00007 10514023

[B70] Haupt S. Berger M. Goldberg Z. Haupt Y. (2003). Apoptosis-the P53 Network. J. Cel. Sci. 116 (20), 4077–4085. 10.1242/jcs.00739 12972501

[B71] He Z.-Y. Shi C.-B. Wen H. Li F.-L. Wang B.-L. Wang J. (2011). Upregulation of P53 Expression in Patients with Colorectal Cancer by Administration of Curcumin. Cancer Invest. 29 (3), 208–213. 10.3109/07357907.2010.550592 21314329

[B72] Heinonen O. P. Koss L. Albanes D. Taylor P. R. Hartman A. M. Edwards B. K. (1998). Prostate Cancer and Supplementation with α-tocopherol and β-carotene: Incidence and Mortality in a Controlled Trial. JNCI: J. Natl. Cancer Inst. 90 (6), 440–446. 10.1093/jnci/90.6.440 9521168

[B73] Hu C. Li M. Guo T. Wang S. Huang W. Yang K. (2019). Anti-metastasis Activity of Curcumin against Breast Cancer via the Inhibition of Stem Cell-like Properties and EMT. Phytomedicine 58, 152740. 10.1016/j.phymed.2018.11.001 31005718

[B74] Huang F.-J. Hsuuw Y.-D. Chan W.-H. (2013). Characterization of Apoptosis Induced by Emodin and Related Regulatory Mechanisms in Human Neuroblastoma Cells. Int. J. Mol. Sci. 14 (10), 20139–20156. 10.3390/ijms141020139 24113589 PMC3821607

[B75] Huang H.-C. Chang J.-H. Tung S.-F. Wu R.-T. Foegh M. L. Chu S.-H. (1992). Immunosuppressive Effect of Emodin, a Free Radical Generator. Eur. J. Pharmacol. 211 (3), 359–364. 10.1016/0014-2999(92)90393-i 1535596

[B76] Huang Q. Shen H.-M. Ong C.-N. (2004). Inhibitory Effect of Emodin on Tumor Invasion through Suppression of Activator Protein-1 and Nuclear Factor-Κb. Biochem. Pharmacol. 68 (2), 361–371. 10.1016/j.bcp.2004.03.032 15194008

[B77] Huang Q. Shen H.-M. Shui G. Wenk M. R. Ong C.-N. (2006). Emodin Inhibits Tumor Cell Adhesion through Disruption of the Membrane Lipid Raft-Associated Integrin Signaling Pathway. Cancer Res. 66 (11), 5807–5815. 10.1158/0008-5472.CAN-06-0077 16740720

[B78] Huang W.-W. Tsai S.-C. Peng S.-F. Lin M.-W. Chiang J.-H. Chiu Y.-J. (2013). Kaempferol Induces Autophagy through AMPK and AKT Signaling Molecules and Causes G2/M Arrest via Downregulation of CDK1/cyclin B in SK-HEP-1 Human Hepatic Cancer Cells. Int. J. Oncol. 42 (6), 2069–2077. 10.3892/ijo.2013.1909 23591552

[B79] I Sakkas L. Mavropoulos A. P Bogdanos D. (2017). Phosphodiesterase 4 Inhibitors in Immune-Mediated Diseases: Mode of Action, Clinical Applications, Current and Future Perspectives. Curr. Med. Chem. 24 (28), 3054–3067. 10.2174/0929867324666170530093902 28554321

[B80] Ingle J. N. Liu M. Wickerham D. L. Schaid D. J. Wang L. Mushiroda T. (2013). Selective Estrogen Receptor Modulators and Pharmacogenomic Variation in ZNF423 Regulation of BRCA1 Expression: Individualized Breast Cancer Prevention. Cancer Discov. 3 (7), 812–825. 10.1158/2159-8290.CD-13-0038 23764426 PMC3710533

[B81] Ishihara T. Kohno K. Ushio S. Iwaki K. Ikeda M. Kurimoto M. (2000). Tryptanthrin Inhibits Nitric Oxide and Prostaglandin E2 Synthesis by Murine Macrophages. Eur. J. Pharmacol. 407 (1-2), 197–204. 10.1016/s0014-2999(00)00674-9 11050308

[B82] Javvadi P. Segan A. T. Tuttle S. W. Koumenis C. (2008). The Chemopreventive Agent Curcumin Is a Potent Radiosensitizer of Human Cervical Tumor Cells via Increased Reactive Oxygen Species Production and Overactivation of the Mitogen-Activated Protein Kinase Pathway. Mol. Pharmacol. 73 (5), 1491–1501. 10.1124/mol.107.043554 18252805 PMC3400533

[B83] Jeong C.-H. Bode A. M. Pugliese A. Cho Y.-Y. Kim H.-G. Shim J.-H. (2009). [6]-Gingerol Suppresses colon Cancer Growth by Targeting Leukotriene A4 Hydrolase. Cancer Res. 69 (13), 5584–5591. 10.1158/0008-5472.CAN-09-0491 19531649

[B84] Jeong J.-W. Lee H. H. Han M. H. Kim G.-Y. Kim W.-J. Choi Y. H. (2014). Anti-inflammatory Effects of Genistein via Suppression of the Toll-like Receptor 4-mediated Signaling Pathway in Lipopolysaccharide-Stimulated BV2 Microglia. Chemico-biological interactions 212, 30–39. 10.1016/j.cbi.2014.01.012 24491678

[B85] Jeong J. C. Kim M. S. Kim T. H. Kim Y. K. (2009). Kaempferol Induces Cell Death through ERK and Akt-dependent Down-Regulation of XIAP and Survivin in Human Glioma Cells. Neurochem. Res. 34 (5), 991–1001. 10.1007/s11064-008-9868-5 18949556

[B86] Ji Q. Liu X. Fu X. Zhang L. Sui H. Zhou L. (2013). Resveratrol Inhibits Invasion and Metastasis of Colorectal Cancer Cells via MALAT1 Mediated Wnt/β-Catenin Signal Pathway. PloS one 8 (11), e78700. 10.1371/journal.pone.0078700 24244343 PMC3823921

[B87] Jiao G. Yan L. Qiang L. Xia G. Ling G. Gui Z. (2013). Resveratrol Induces Apoptosis and Autophagy in T-Cell Acute Lymphoblastic Leukemia Cells by Inhibiting Akt/mTOR and Activating P38-MAPK. Biomed. Environ. Sci. 26 (11), 902–911. 10.3967/bes2013.019 24331535

[B88] Johnson J. J. Nihal M. Siddiqui I. A. Scarlett C. O. Bailey H. H. Mukhtar H. (2011). Enhancing the Bioavailability of Resveratrol by Combining it with Piperine. Mol. Nutr. Food Res. 55 (8), 1169–1176. 10.1002/mnfr.201100117 21714124 PMC3295233

[B89] Joo J.-H. Hong S.-S. Cho Y.-R. Seo D.-W. (2016). 10-Gingerol Inhibits Proliferation and Invasion of MDA-MB-231 Breast Cancer Cells through Suppression of Akt and p38MAPK Activity. Oncol. Rep. 35 (2), 779–784. 10.3892/or.2015.4405 26554741

[B90] Jun K. Y. Park S. E. Liang J. L. Jahng Y. Kwon Y. (2015). Benzo [b] Tryptanthrin Inhibits MDR1, Topoisomerase Activity, and Reverses Adriamycin Resistance in Breast Cancer Cells. ChemMedChem 10 (5), 827–835. 10.1002/cmdc.201500068 25809558

[B91] Kadioglu O. Nass J. Saeed M. E. Schuler B. Efferth T. (2015). Kaempferol Is an Anti-inflammatory Compound with Activity towards NF-Κb Pathway Proteins. Anticancer Res. 35 (5), 2645–2650. 25964540

[B92] Kang S. Kim B. R. Kang M.-H. Kim D.-Y. Lee D.-H. Oh S. C. (2018). Anti-metastatic Effect of Metformin via Repression of Interleukin 6-induced Epithelial–Mesenchymal Transition in Human colon Cancer Cells. PloS one 13 (10), e0205449. 10.1371/journal.pone.0205449 30308035 PMC6181375

[B93] Kavanagh K. T. Hafer L. J. Kim D. W. Mann K. K. Sherr D. H. Rogers A. E. (2001). Green tea Extracts Decrease Carcinogen‐induced Mammary Tumor burden in Rats and Rate of Breast Cancer Cell Proliferation in Culture. J. Cell. Biochem. 82 (3), 387–398. 10.1002/jcb.1164 11500915

[B94] Keith R. L. Blatchford P. J. Merrick D. T. Bunn P. A. Bagwell B. Dwyer-Nield L. D. (2019). A Randomized Phase II Trial of Pioglitazone for Lung Cancer Chemoprevention in High-Risk Current and Former Smokers. Cancer Prev. Res. 12 (10), 721–730. 10.1158/1940-6207.CAPR-19-0006 PMC6774885 31308004

[B95] Khandrika L. Kumar B. Koul S. Maroni P. Koul H. K. (2009). Oxidative Stress in Prostate Cancer. Cancer Lett. 282 (2), 125–136. 10.1016/j.canlet.2008.12.011 19185987 PMC2789743

[B96] Kim A. L. Zhu Y. Zhu H. Han L. Kopelovich L. Bickers D. R. (2006). Resveratrol Inhibits Proliferation of Human Epidermoid Carcinoma A431 Cells by Modulating MEK1 and AP‐1 Signalling Pathways. Exp. Dermatol. 15 (7), 538–546. 10.1111/j.1600-0625.2006.00445.x 16761963

[B97] Kim B.-W. Lee E.-R. Min H.-M. Jeong H.-S. Ahn J.-Y. Kim J.-H. (2008). Sustained ERK Activation Is Involved in the Kaempferol-Induced Apoptosis of Breast Cancer Cells and Is More Evident under 3-D Culture Condition. Cancer Biol. Ther. 7 (7), 1080–1089. 10.4161/cbt.7.7.6164 18443432

[B98] Kim S. G. Veena M. S. Basak S. K. Han E. Tajima T. Gjertson D. W. (2011). Curcumin Treatment Suppresses IKKβ Kinase Activity of Salivary Cells of Patients with Head and Neck Cancer: a Pilot Study. Clin. Cancer Res. 17 (18), 5953–5961. 10.1158/1078-0432.CCR-11-1272 21821700 PMC3176971

[B99] Kim S. O. Kundu J. K. Shin Y. K. Park J.-H. Cho M.-H. Kim T.-Y. (2005). [6]-Gingerol Inhibits COX-2 Expression by Blocking the Activation of P38 MAP Kinase and NF-κ B in Phorbol Ester-Stimulated Mouse Skin. Oncogene 24 (15), 2558–2567. 10.1038/sj.onc.1208446 15735738

[B100] Kim T. W. Lee S. Y. Kim M. Cheon C. Ko S.-G. (2018). Kaempferol Induces Autophagic Cell Death via IRE1-JNK-CHOP Pathway and Inhibition of G9a in Gastric Cancer Cells. Cel Death Dis. 9 (9), 1–14. 10.1038/s41419-018-0930-1 PMC6115440 30158521

[B101] Kimoto T. Hino K. Koya‐Miyata S. Yamamoto Y. Takeuchi M. Nishizaki Y. (2001). Cell Differentiation and Apoptosis of Monocytic and Promyelocytic Leukemia Cells (U‐937 and HL‐60) by Tryptanthrin, an Active Ingredient of Polygonum Tinctorium Lour. Pathol. Int. 51 (5), 315–325. 10.1046/j.1440-1827.2001.01204.x 11422788

[B102] Klein E. A. Thompson I. M. Tangen C. M. Crowley J. J. Lucia M. S. Goodman P. J. (2011). Vitamin E and the Risk of Prostate Cancer: the Selenium and Vitamin E Cancer Prevention Trial (SELECT). Jama 306 (14), 1549–1556. 10.1001/jama.2011.1437 21990298 PMC4169010

[B103] Kores K. Lesnik S. Bren U. Janezic D. Konc J. (2019). Discovery of Novel Potential Human Targets of Resveratrol by Inverse Molecular Docking. J. Chem. Inf. Model. 59 (5), 2467–2478. 10.1021/acs.jcim.8b00981 30883115

[B104] Koya-Miyata S. Kimoto T. Micallef M. J. Hino K. Taniguchi M. Ushio S. (2001). Prevention of Azoxymethane-Induced Intestinal Tumors by a Crude Ethyl Acetate-Extract and Tryptanthrin Extracted from Polygonum Tinctorium Lour. Anticancer Res. 21 (5), 3295–3300. 11848486

[B105] Koyama J. Morita I. Tagahara K. Nobukuni Y. Mukainaka T. Kuchide M. (2002). Chemopreventive Effects of Emodin and Cassiamin B in Mouse Skin Carcinogenesis. Cancer Lett. 182 (2), 135–139. 10.1016/s0304-3835(02)00100-3 12048158

[B106] Kumara M. Shylajab M. Nazeemc P. Babu T. (2017). 6-Gingerol Is the Most Potent Anticancerous Compound in Ginger (Zingiber Officinale Rosc.). J. Developing Drugs 6 (1), 1–6. 10.4172/2329-6631.1000167

[B107] Kuttan R. Sudheeran P. Josph C. (1987). Turmeric and Curcumin as Topical Agents in Cancer Therapy. Tumori J. 73 (1), 29–31. 10.1177/030089168707300105 2435036

[B108] Langová M. Polívková Z. Šmerák P. Bártová J. Barta I. (2005). Antimutagenic Effect of Resveratrol. Czech J. Food Sci. 23 (5), 202.

[B109] Lee H. S. Cho H. J. Yu R. Lee K. W. Chun H. S. Park J. H. Y. (2014). Mechanisms Underlying Apoptosis-Inducing Effects of Kaempferol in HT-29 Human colon Cancer Cells. Int. J. Mol. Sci. 15 (2), 2722–2737. 10.3390/ijms15022722 24549175 PMC3958878

[B110] Lee H. Tsai S.-J. (1991). Effect of Emodin on Cooked-Food Mutagen Activation. Food Chem. Toxicol. 29 (11), 765–770. 10.1016/0278-6915(91)90185-a 1761256

[B111] Lee H. Z. (2001). Protein Kinase C Involvement in Aloe‐emodin‐and Emodin‐induced Apoptosis in Lung Carcinoma Cell. Br. J. Pharmacol. 134 (5), 1093–1103. 10.1038/sj.bjp.0704342 11682458 PMC1573035

[B112] Lee J. Kim J. H. (2016). Kaempferol Inhibits Pancreatic Cancer Cell Growth and Migration through the Blockade of EGFR-Related Pathway *In Vitro* . PloS one 11 (5), e0155264. 10.1371/journal.pone.0155264 27175782 PMC4866780

[B113] Lee S. R. Kwon S. W. Lee Y. H. Kaya P. Kim J. M. Ahn C. (2019). Dietary Intake of Genistein Suppresses Hepatocellular Carcinoma through AMPK-Mediated Apoptosis and Anti-inflammation. BMC cancer 19 (1), 1–12. 10.1186/s12885-018-5222-8 30606143 PMC6318960

[B114] Leiter U. Garbe C. (2008). Epidemiology of Melanoma and Nonmelanoma Skin Cancer—The Role of Sunlight. Adv. Exp. Med. Biol. 624, 89–103. 10.1007/978-0-387-77574-6_8 18348450

[B115] Li L.-L. Cui Y. Guo X.-H. Ma K. Tian P. Feng J. (2019). Pharmacokinetics and Tissue Distribution of Gingerols and Shogaols from Ginger (Zingiber Officinale rosc.) in Rats by UPLC–Q-Exactive–HRMS. Molecules 24 (3), 512. 10.3390/molecules24030512 PMC6384666 30708987

[B116] Li Y. Sarkar F. H. (2002). Inhibition of Nuclear Factor κB Activation in PC3 Cells by Genistein Is Mediated via Akt Signaling Pathway. Clin. Cancer Res. 8 (7), 2369–2377. 12114442

[B117] Li Y. Wu L. Li K. Liu Y. Xiang R. Zhang S. (2011). Involvement of Nuclear Factor κB (NF-Κb) in the Downregulation of Cyclo-Oxygenase-2 (COX-2) by Genistein in Gastric Cancer Cells. J. Int. Med. Res. 39 (6), 2141–2150. 10.1177/147323001103900610 22289529

[B118] Liang T. Zhang X. Xue W. Zhao S. Zhang X. Pei J. (2014). Curcumin Induced Human Gastric Cancer BGC-823 Cells Apoptosis by ROS-Mediated ASK1-MKK4-JNK Stress Signaling Pathway. Int. J. Mol. Sci. 15 (9), 15754–15765. 10.3390/ijms150915754 25198898 PMC4200840

[B119] Liao W. Xiang W. Wang F.-F. Wang R. Ding Y. (2017). Curcumin Inhibited Growth of Human Melanoma A375 Cells via Inciting Oxidative Stress. Biomed. Pharmacother. 95, 1177–1186. 10.1016/j.biopha.2017.09.026 28926928

[B120] Liao X. Leung K. N. (2013). Tryptanthrin Induces Growth Inhibition and Neuronal Differentiation in the Human Neuroblastoma LA-N-1 Cells. Chemico-biological interactions 203 (2), 512–521. 10.1016/j.cbi.2013.03.001 23500671

[B121] Liao X. Zhou X. Mak N.-k. Leung K.-n. (2013). Tryptanthrin Inhibits Angiogenesis by Targeting the VEGFR2-Mediated ERK1/2 Signalling Pathway. Plos one 8 (12), e82294. 10.1371/journal.pone.0082294 24358167 PMC3864955

[B122] Lin C.-W. Chen P.-N. Chen M.-K. Yang W.-E. Tang C.-H. Yang S.-F. (2013). Kaempferol Reduces Matrix Metalloproteinase-2 Expression by Down-Regulating ERK1/2 and the Activator Protein-1 Signaling Pathways in Oral Cancer Cells. PloS one 8 (11), e80883. 10.1371/journal.pone.0080883 24278338 PMC3835430

[B123] Lippman S. M. Klein E. A. Goodman P. J. Lucia M. S. Thompson I. M. Ford L. G. (2009). Effect of Selenium and Vitamin E on Risk of Prostate Cancer and Other Cancers: the Selenium and Vitamin E Cancer Prevention Trial (SELECT). Jama 301 (1), 39–51. 10.1001/jama.2008.864 19066370 PMC3682779

[B124] Liu D.-m. Yang D. Zhou C.-y. Wu J.-s. Zhang G.-l. Wang P. (2020). Aloe-emodin Induces Hepatotoxicity by the Inhibition of Multidrug Resistance Protein 2. Phytomedicine 68, 153148. 10.1016/j.phymed.2019.153148 32028185

[B125] Liu H. Yu G. (2004). Antimetastatic Effects of Genistein on Salivary Adenoid Cystic Carcinoma *In Vivo* . Zhonghua Kou Qiang Yi Xue Za Zhi 39 (5), 373–375. 15498340

[B126] Liu Z. Wu X. Lv J. Sun H. Zhou F. (2019). Resveratrol Induces P53 in Colorectal Cancer through SET7/9. Oncol. Lett. 17 (4), 3783–3789. 10.3892/ol.2019.10034 30881498 PMC6403518

[B127] Lo J. J. Park Y. M. M. Sinha R. Sandler D. P. (2020). Association between Meat Consumption and Risk of Breast Cancer: Findings from the Sister Study. Int. J. Cancer 146 (8), 2156–2165. 10.1002/ijc.32547 31389007 PMC7002279

[B128] Lohse I. Wildermuth E. Brothers S. P. (2018). Naturally Occurring Compounds as Pancreatic Cancer Therapeutics. Oncotarget 9 (83), 35448. 10.18632/oncotarget.26234 30459936 PMC6226042

[B129] Lomas A. Leonardi‐Bee J. Bath‐Hextall F. (2012). A Systematic Review of Worldwide Incidence of Nonmelanoma Skin Cancer. Br. J. Dermatol. 166 (5), 1069–1080. 10.1111/j.1365-2133.2012.10830.x 22251204

[B130] Luo H. Jiang B. Li B. Li Z. Jiang B.-H. Chen Y. C. (2012). Kaempferol Nanoparticles Achieve strong and Selective Inhibition of Ovarian Cancer Cell Viability. Int. J. nanomedicine 7, 3951. 10.2147/IJN.S33670 22866004 PMC3410694

[B131] Luo H. Rankin G. O. Juliano N. Jiang B.-H. Chen Y. C. (2012). Kaempferol Inhibits VEGF Expression and *In Vitro* Angiogenesis through a Novel ERK-Nfκb-cMyc-P21 Pathway. Food Chem. 130 (2), 321–328. 10.1016/j.foodchem.2011.07.045 21927533 PMC3171974

[B132] Luo H. Rankin G. O. Liu L. Daddysman M. K. Jiang B.-H. Chen Y. C. (2009). Kaempferol Inhibits Angiogenesis and VEGF Expression through Both HIF Dependent and Independent Pathways in Human Ovarian Cancer Cells. Nutr. Cancer 61 (4), 554–563. 10.1080/01635580802666281 19838928 PMC2770884

[B133] Malloy K. M. Wang J. Clark L. H. Fang Z. Sun W. Yin Y. (2018). Novasoy and Genistein Inhibit Endometrial Cancer Cell Proliferation through Disruption of the AKT/mTOR and MAPK Signaling Pathways. Am. J. translational Res. 10 (3), 784. PMC5883119 29636868

[B134] Mao J. T. Xue B. Fan S. Neis P. Qualls C. Massie L. (2021). Leucoselect Phytosome Modulates Serum Eicosapentaenoic Acid, Docosahexaenoic Acid, and Prostaglandin E3 in a Phase I Lung Cancer Chemoprevention Study. Cancer Prev. Res. 14 (6), 619–626. 10.1158/1940-6207.CAPR-20-0585 PMC8225569 33707173

[B135] Mao J. T. Xue B. Smoake J. Lu Q.-Y. Park H. Henning S. M. (2016). MicroRNA-19a/b Mediates Grape Seed Procyanidin Extract-Induced Anti-neoplastic Effects against Lung Cancer. J. Nutr. Biochem. 34, 118–125. 10.1016/j.jnutbio.2016.05.003 27289489 PMC8152178

[B136] Maria McCrohan A. Morrissey C. O'Keane C. Mulligan N. Watson C. Smith J. (2006). Effects of the Dual 5 α‐reductase Inhibitor Dutasteride on Apoptosis in Primary Cultures of Prostate Cancer Epithelial Cells and Cell Lines. Cancer Interdiscip. Int. J. Am. Cancer Soc. 106 (12), 2743–2752. 10.1002/cncr.21938 16703599

[B137] Marks R. Rennie G. Selwood T. (1988). Malignant Transformation of Solar Keratoses to Squamous Cell Carcinoma. The Lancet 331 (8589), 795–797. 10.1016/s0140-6736(88)91658-3 2895318

[B138] Marquardt J. U. Gomez-Quiroz L. Camacho L. O. A. Pinna F. Lee Y.-H. Kitade M. (2015). Curcumin Effectively Inhibits Oncogenic NF-Κb Signaling and Restrains Stemness Features in Liver Cancer. J. Hepatol. 63 (3), 661–669. 10.1016/j.jhep.2015.04.018 25937435 PMC4543531

[B139] Martin A. C. B. M. Tomasin R. Luna-Dulcey L. Graminha A. E. Naves M. A. Teles R. H. G. (2020). [10]-Gingerol Improves Doxorubicin Anticancer Activity and Decreases its Side Effects in Triple Negative Breast Cancer Models. Cell Oncol. 43 (5), 915–929. 10.1007/s13402-020-00539-z PMC12990714 32761561

[B140] Martinez E. E. Darke A. K. Tangen C. M. Goodman P. J. Fowke J. H. Klein E. A. (2014). A Functional Variant in NKX3. 1 Associated with Prostate Cancer Risk in the Selenium and Vitamin E Cancer Prevention Trial (SELECT). Cancer Prev. Res. 7 (9), 950–957. 10.1158/1940-6207.CAPR-14-0075 PMC4154984 24894197

[B141] Miao S. Shi X. Zhang H. Wang S. Sun J. Hua W. (2011). Proliferation-attenuating and Apoptosis-Inducing Effects of Tryptanthrin on Human Chronic Myeloid Leukemia K562 Cell Line *In Vitro* . Int. J. Mol. Sci. 12 (6), 3831–3845. 10.3390/ijms12063831 21747710 PMC3131594

[B142] Monteillier A. Voisin A. Furrer P. Allémann E. Cuendet M. (2018). Intranasal Administration of Resveratrol Successfully Prevents Lung Cancer in A/J Mice. Scientific Rep. 8 (1), 1–9. 10.1038/s41598-018-32423-0 PMC6155121 30250304

[B143] Morse M. A. Wang C.-X. Stoner G. D. Mandal S. Conran P. B. Amin S. G. (1989). Inhibition of 4-(methylnitrosamino)-1-(3-Pyridyl)-1-Butanone-Induced DNA Adduct Formation and Tumorigenicity in the Lung of F344 Rats by Dietary Phenethyl Isothiocyanate. Cancer Res. 49 (3), 549–553. 2910476

[B144] Mouria M. Gukovskaya A. S. Jung Y. Buechler P. Hines O. J. Reber H. A. (2002). Food‐derived Polyphenols Inhibit Pancreatic Cancer Growth through Mitochondrial Cytochrome C Release and Apoptosis. Int. J. Cancer 98 (5), 761–769. 10.1002/ijc.10202 11920648

[B145] Mukund V. Behera S. K. Alam A. Nagaraju G. P. (2019). Molecular Docking Analysis of Nuclear Factor-Κb and Genistein Interaction in the Context of Breast Cancer. Bioinformation 15 (1), 11. 10.6026/97320630015011 31359993 PMC6651031

[B146] Mukund V. (2020). Genistein: Its Role in Breast Cancer Growth and Metastasis. Curr. Drug Metab. 21 (1), 6–10. 10.2174/1389200221666200120121919 31987018

[B147] Mutlu Altundağ E. Yılmaz A. M. Serdar B. S. Jannuzzi A. T. Koçtürk S. Yalçın A. S. (2021). Synergistic Induction of Apoptosis by Quercetin and Curcumin in Chronic Myeloid Leukemia (K562) Cells: II. Signal Transduction Pathways Involved. Nutr. Cancer 73 (4), 703–712. 10.1080/01635581.2020.1767167 32420759

[B148] Muto A. Hori M. Sasaki Y. Saitoh A. Yasuda I. Maekawa T. (2007). Emodin Has a Cytotoxic Activity against Human Multiple Myeloma as a Janus-Activated Kinase 2 Inhibitor. Mol. Cancer Ther. 6 (3), 987–994. 10.1158/1535-7163.MCT-06-0605 17363492

[B149] Mylonis I. Lakka A. Tsakalof A. Simos G. (2010). The Dietary Flavonoid Kaempferol Effectively Inhibits HIF-1 Activity and Hepatoma Cancer Cell Viability under Hypoxic Conditions. Biochem. biophysical Res. Commun. 398 (1), 74–78. 10.1016/j.bbrc.2010.06.038 20558139

[B150] Nair H. H. Alex V. V. Anto R. J. (2021). Significance of Nutraceuticals in Cancer Therapy. Evolutionary Diversity as a Source for Anticancer Molecules. Elsevier, 309–321. 10.1016/b978-0-12-821710-8.00014-x

[B151] Nair K. L. Thulasidasan A. K. T. Deepa G. Anto R. J. Kumar G. V. (2012). Purely Aqueous PLGA Nanoparticulate Formulations of Curcumin Exhibit Enhanced Anticancer Activity with Dependence on the Combination of the Carrier. Int. J. pharmaceutics 425 (1-2), 44–52. 10.1016/j.ijpharm.2012.01.003 22266528

[B152] Nair M. P. Kandaswami C. Mahajan S. Chadha K. C. Chawda R. Nair H. (2002). The Flavonoid, Quercetin, Differentially Regulates Th-1 (IFNγ) and Th-2 (IL4) Cytokine Gene Expression by normal Peripheral Blood Mononuclear Cells. Biochim. Biophys. Acta (BBA)-Molecular Cel Res. 1593 (1), 29–36. 10.1016/s0167-4889(02)00328-2 12431781

[B153] Nakajima M. Yoshida R. Shimada N. Yamazaki H. Yokoi T. (2001). Inhibition and Inactivation of Human Cytochrome P450 Isoforms by Phenethyl Isothiocyanate. Drug Metab. Disposition 29 (8), 1110–1113. 11454729

[B154] Nath L. R. Gorantla J. N. Joseph S. M. Antony J. Thankachan S. Menon D. B. (2015). Kaempferide, the Most Active Among the Four Flavonoids Isolated and Characterized from Chromolaena Odorata, Induces Apoptosis in Cervical Cancer Cells while Being Pharmacologically Safe. RSC Adv. 5 (122), 100912–100922. 10.1039/c5ra19199h

[B155] Nguyen T. Tran E. Ong C. Lee S. Do P. Huynh T. (2003). Kaempferol‐induced Growth Inhibition and Apoptosis in A549 Lung Cancer Cells Is Mediated by Activation of MEK‐MAPK. J. Cell. Physiol. 197 (1), 110–121. 10.1002/jcp.10340 12942547

[B156] Nigam N. George J. Srivastava S. Roy P. Bhui K. Singh M. (2010). Induction of Apoptosis by [6]-gingerol Associated with the Modulation of P53 and Involvement of Mitochondrial Signaling Pathway in B [a] P-Induced Mouse Skin Tumorigenesis. Cancer Chemother. Pharmacol. 65 (4), 687–696. 10.1007/s00280-009-1074-x 19629484

[B157] Niu Y.-N. Wang K. Jin S. Fan D.-D. Wang M.-S. Xing N.-Z. (2016). The Intriguing Role of Fibroblasts and C-Jun in the Chemopreventive and Therapeutic Effect of Finasteride on Xenograft Models of Prostate Cancer. Asian J. Androl. 18 (6), 913. 10.4103/1008-682X.167714 26698232 PMC5109888

[B158] O'riordan J. Abdel-Latif M. Ravi N. McNamara D. Byrne P. McDonald G. (2005). Proinflammatory Cytokine and Nuclear Factor Kappa-B Expression along the Inflammation–Metaplasia–Dysplasia–Adenocarcinoma Sequence in the Esophagus. Official J. Am. Coll. Gastroenterol. ACG 100 (6), 1257–1264. 10.1111/j.1572-0241.2005.41338.x 15929754

[B159] Olsen B. B. Bjørling-Poulsen M. Guerra B. (2007). Emodin Negatively Affects the Phosphoinositide 3-kinase/AKT Signalling Pathway: a Study on its Mechanism of Action. Int. J. Biochem. Cel. Biol. 39 (1), 227–237. 10.1016/j.biocel.2006.08.006 17018259

[B160] Omenn G. S. Goodman G. E. Thornquist M. D. Balmes J. Cullen M. R. Glass A. (1996). Effects of a Combination of Beta Carotene and Vitamin A on Lung Cancer and Cardiovascular Disease. New Engl. J. Med. 334 (18), 1150–1155. 10.1056/NEJM199605023341802 8602180

[B161] Ozkan E. E. (2011). Plasma and Tissue Insulin-like Growth Factor-I Receptor (IGF-IR) as a Prognostic Marker for Prostate Cancer and Anti-IGF-IR Agents as Novel Therapeutic Strategy for Refractory Cases: a Review. Mol. Cell. Endocrinol. 344 (1-2), 1–24. 10.1016/j.mce.2011.07.002 21782884

[B162] Pan H. Zhou W. He W. Liu X. Ding Q. Ling L. (2012). Genistein Inhibits MDA-MB-231 Triple-Negative Breast Cancer Cell Growth by Inhibiting NF-Κb Activity via the Notch-1 Pathway. Int. J. Mol. Med. 30 (2), 337–343. 10.3892/ijmm.2012.990 22580499

[B163] Pang X. Fu W. Wang J. Kang D. Xu L. Zhao Y. (2018). Dentification of Estrogen Receptor α Antagonists from Natural Products via *In Vitro* and In Silico approaches. Oxidative Med. Cell. longevity 2018. 10.1155/2018/6040149 I PMC5971309 29861831

[B164] Pathania A. S. Kumar S. Guru S. K. Bhushan S. Sharma P. R. Aithagani S. K. (2014). The Synthetic Tryptanthrin Analogue Suppresses STAT3 Signaling and Induces Caspase Dependent Apoptosis via ERK up Regulation in Human Leukemia HL-60 Cells. Plos one 9 (11), e110411. 10.1371/journal.pone.0110411 25383546 PMC4226462

[B165] Pavan A. R. Silva G. D. B. d. Jornada D. H. Chiba D. E. Fernandes G. F. d. S. Man Chin C. (2016). Unraveling the Anticancer Effect of Curcumin and Resveratrol. Nutrients 8 (11), 628. 10.3390/nu8110628 PMC5133053 27834913

[B166] Pentland A. P. Schoggins J. W. Scott G. A. Khan K. N. M. Han R. (1999). Reduction of UV-Induced Skin Tumors in Hairless Mice by Selective COX-2 Inhibition. Carcinogenesis 20 (10), 1939–1944. 10.1093/carcin/20.10.1939 10506108

[B167] Polivkova Z. Langova M. Smerak P. Bartova J. Barta I. (2006). Antimutagenic Effect of Genistein. Czech J. Food Sci. 24 (3), 119.

[B168] Pratheeshkumar P. Budhraja A. Son Y.-O. Wang X. Zhang Z. Ding S. (2012). "Quercetin Inhibits Angiogenesis Mediated Human Prostate Tumor Growth by Targeting VEGFR-2 Regulated AKT/mTOR/P70S6K Signaling Pathways." 10.1371/journal.pone.0047516 PMC3475699 23094058

[B169] Pricci M. Girardi B. Giorgio F. Losurdo G. Ierardi E. Di Leo A. (2020). Curcumin and Colorectal Cancer: from Basic to Clinical Evidences. Int. J. Mol. Sci. 21 (7), 2364. 10.3390/ijms21072364 PMC7178200 32235371

[B170] Priyadarsini R. V. Murugan R. S. Maitreyi S. Ramalingam K. Karunagaran D. Nagini S. (2010). The Flavonoid Quercetin Induces Cell Cycle Arrest and Mitochondria-Mediated Apoptosis in Human Cervical Cancer (HeLa) Cells through P53 Induction and NF-Κb Inhibition. Eur. J. Pharmacol. 649 (1-3), 84–91. 10.1016/j.ejphar.2010.09.020 20858478

[B171] Puliyappadamba V. T. Cheriyan V. T. Thulasidasan A. K. T. Bava S. V. Vinod B. S. Prabhu P. R. (2010). Nicotine-induced Survival Signaling in Lung Cancer Cells Is Dependent on Their P53 Status while its Down-Regulation by Curcumin Is Independent. Mol. Cancer 9 (1), 1–15. 10.1186/1476-4598-9-220 20727180 PMC2936340

[B172] Puliyappadamba V. T. Thulasidasan A. K. T. Vijayakurup V. Antony J. Bava S. V. Anwar S. (2015). Curcumin Inhibits B [a] PDE‐induced Procarcinogenic Signals in Lung Cancer Cells, and Curbs B [a] P‐induced Mutagenesis and Lung Carcinogenesis. BioFactors 41 (6), 431–442. 10.1002/biof.1244 26643788

[B173] Qin Q.-P. Zou B.-Q. Tan M.-X. Wang S.-L. Liu Y.-C. Liang H. (2018). Tryptanthrin Derivative Copper (Ii) Complexes with High Antitumor Activity by Inhibiting Telomerase Activity, and Inducing Mitochondria-Mediated Apoptosis and S-phase Arrest in BEL-7402. New J. Chem. 42 (18), 15479–15487. 10.1039/c8nj03005g

[B174] Qiu P. Zhang S. Zhou Y. Zhu M. Kang Y. Chen D. (2017). Synthesis and Evaluation of Asymmetric Curcuminoid Analogs as Potential Anticancer Agents that Downregulate NF-Κb Activation and Enhance the Sensitivity of Gastric Cancer Cell Lines to Irinotecan Chemotherapy. Eur. J. Med. Chem. 139, 917–925. 10.1016/j.ejmech.2017.08.022 28881286

[B175] Radhakrishnan E. Bava S. V. Narayanan S. S. Nath L. R. Thulasidasan A. K. T. Soniya E. V. (2014). [6]-Gingerol Induces Caspase-dependent Apoptosis and Prevents PMA-Induced Proliferation in colon Cancer Cells by Inhibiting MAPK/AP-1 Signaling. PloS one 9 (8), e104401. 10.1371/journal.pone.0104401 25157570 PMC4144808

[B176] Rafiq R. A. Quadri A. Nazir L. A. Peerzada K. Ganai B. A. Tasduq S. A. (2015). A Potent Inhibitor of Phosphoinositide 3-Kinase (PI3K) and Mitogen Activated Protein (MAP) Kinase Signalling, Quercetin (3, 3', 4', 5, 7-Pentahydroxyflavone) Promotes Cell Death in Ultraviolet (UV)-B-irradiated B16F10 Melanoma Cells. PloS one 10 (7), e0131253. 10.1371/journal.pone.0131253 26148186 PMC4493061

[B177] Rashid A. Liu C. Sanli T. Tsiani E. Singh G. Bristow R. G. (2011). Resveratrol Enhances Prostate Cancer Cell Response to Ionizing Radiation. Modulation of the AMPK, Akt and mTOR Pathways. Radiat. Oncol. 6 (1), 1–12. 10.1186/1748-717X-6-144 22029423 PMC3217881

[B178] Rather R. A. Bhagat M. (2018). Cancer Chemoprevention and Piperine: Molecular Mechanisms and Therapeutic Opportunities. Front. Cel. Dev. Biol. 6, 10. 10.3389/fcell.2018.00010 PMC5818432 29497610

[B179] Rawat D. Chhonker S. K. Naik R. A. Koiri R. K. (2021). Modulation of Antioxidant Enzymes, SIRT1 and NF‐κB by Resveratrol and Nicotinamide in Alcohol‐aflatoxin B1‐induced Hepatocellular Carcinoma. J. Biochem. Mol. Toxicol. 35 (1), e22625. 10.1002/jbt.22625 32894639

[B180] Ray A. Vasudevan S. Sengupta S. (2015). 6-Shogaol Inhibits Breast Cancer Cells and Stem Cell-like Spheroids by Modulation of Notch Signaling Pathway and Induction of Autophagic Cell Death. PloS one 10 (9), e0137614. 10.1371/journal.pone.0137614 26355461 PMC4565635

[B181] Riahi-Chebbi I. Souid S. Othman H. Haoues M. Karoui H. Morel A. (2019). The Phenolic Compound Kaempferol Overcomes 5-fluorouracil Resistance in Human Resistant LS174 colon Cancer Cells. Scientific Rep. 9 (1), 1–20. 10.1038/s41598-018-36808-z PMC6336835 30655588

[B182] Ro J.-H. Liu C.-C. Lin M.-C. (2021). Resveratrol Mitigates Cerebral Ischemic Injury by Altering Levels of Trace Elements, Toxic Metal, Lipid Peroxidation, and Antioxidant Activity. Biol. Trace Elem. Res. 199 (10), 3718–3727. 10.1007/s12011-020-02497-x 33230635

[B183] Robaszkiewicz A. Balcerczyk A. Bartosz G. (2007). Antioxidative and Prooxidative Effects of Quercetin on A549 Cells. Cel Biol. Int. 31 (10), 1245–1250. 10.1016/j.cellbi.2007.04.009 17583542

[B184] Sabatini M. E. Chiocca S. (2020). Human Papillomavirus as a Driver of Head and Neck Cancers. Br. J. Cancer 122 (3), 306–314. 10.1038/s41416-019-0602-7 31708575 PMC7000688

[B185] Sahin K. Orhan C. Tuzcu M. Sahin N. Tastan H. Özercan İ. H. (2018). Chemopreventive and Antitumor Efficacy of Curcumin in a Spontaneously Developing Hen Ovarian Cancer Model. Cancer Prev. Res. 11 (1), 59–67. 10.1158/1940-6207.CAPR-16-0289 29089332

[B186] Sakalli-Tecim E. Uyar-Arpaci P. Guray N. T. (2021). Identification of Potential Therapeutic Genes and Pathways in Phytoestrogen Emodin Treated Breast Cancer Cell Lines via Network Biology Approaches. Nutr. Cancer, 1–13. 10.1080/01635581.2021.1889622 33645356

[B187] Samavat H. Ursin G. Emory T. H. Lee E. Wang R. Torkelson C. J. (2017). A Randomized Controlled Trial of green tea Extract Supplementation and Mammographic Density in Postmenopausal Women at Increased Risk of Breast Cancer. Cancer Prev. Res. 10 (12), 710–718. 10.1158/1940-6207.CAPR-17-0187 PMC7337967 28904061

[B188] Saw C. L. L. Guo Y. Yang A. Y. Paredes-Gonzalez X. Ramirez C. Pung D. (2014). The berry Constituents Quercetin, Kaempferol, and Pterostilbene Synergistically Attenuate Reactive Oxygen Species: Involvement of the Nrf2-ARE Signaling Pathway. Food Chem. Toxicol. 72, 303–311. 10.1016/j.fct.2014.07.038 25111660

[B189] Schmidt L. J. Regan K. M. Anderson S. K. Sun Z. Ballman K. V. Tindall D. J. (2009). Effects of the 5 Alpha‐reductase Inhibitor Dutasteride on Gene Expression in Prostate Cancer Xenografts. The Prostate 69 (16), 1730–1743. 10.1002/pros.21022 19676081 PMC2783419

[B190] Schütze M. Boeing H. Pischon T. Rehm J. Kehoe T. Gmel G. (2011). Alcohol Attributable burden of Incidence of Cancer in Eight European Countries Based on Results from Prospective Cohort Study. BMJ 342. 10.1136/bmj.d1584 PMC3072472 21474525

[B191] Senthilkumar K. Elumalai P. Arunkumar R. Banudevi S. Gunadharini N. D. Sharmila G. (2010). Quercetin Regulates Insulin like Growth Factor Signaling and Induces Intrinsic and Extrinsic Pathway Mediated Apoptosis in Androgen Independent Prostate Cancer Cells (PC-3). Mol. Cell. Biochem. 344 (1), 173–184. 10.1007/s11010-010-0540-4 20658310

[B192] Shafiee G. Saidijam M. Tavilani H. Ghasemkhani N. Khodadadi I. (2016). Genistein Induces Apoptosis and Inhibits Proliferation of HT29 colon Cancer Cells. Int. J. Mol. Cell. Med. 5 (3), 178. 27942504 PMC5125370

[B193] Shankar G M. Alex V. V. Nisthul A A. Bava S. V. Sundaram S. Retnakumari A. P. (2020). Pre‐clinical Evidences for the Efficacy of Tryptanthrin as a Potent Suppressor of Skin Cancer. Cel Prolif. 53 (1), e12710. 10.1111/cpr.12710 PMC6985671 31663659

[B194] Shankar G. M. Antony J. Anto R. J. (2015). Quercetin and Tryptanthrin: Two Broad Spectrum Anticancer Agents for Future Chemotherapeutic Interventions. The enzymes, 37, 43–72. 10.1016/bs.enz.2015.05.001 26298455

[B195] Sharma V. Joseph C. Ghosh S. Agarwal A. Mishra M. K. Sen E. (2007). Kaempferol Induces Apoptosis in Glioblastoma Cells through Oxidative Stress. Mol. Cancer Ther. 6 (9), 2544–2553. 10.1158/1535-7163.MCT-06-0788 17876051

[B196] Sharmila G. Bhat F. Arunkumar R. Elumalai P. Singh P. R. Senthilkumar K. (2014). Chemopreventive Effect of Quercetin, a Natural Dietary Flavonoid on Prostate Cancer in *In Vivo* Model. Clin. Nutr. 33 (4), 718–726. 10.1016/j.clnu.2013.08.011 24080313

[B197] Shen X. Si Y. Wang Z. Wang J. Guo Y. Zhang X. (2016). Quercetin Inhibits the Growth of Human Gastric Cancer Stem Cells by Inducing Mitochondrial-dependent Apoptosis through the Inhibition of PI3K/Akt Signaling. Int. J. Mol. Med. 38 (2), 619–626. 10.3892/ijmm.2016.2625 27278820

[B198] Shia C.-S. Hou Y.-C. Tsai S.-Y. Huieh P.-H. Leu Y.-L. Chao P.-D. L. (2010). Differences in Pharmacokinetics and *Ex Vivo* Antioxidant Activity Following Intravenous and Oral Administrations of Emodin to Rats. J. Pharm. Sci. 99 (4), 2185–2195. 10.1002/jps.21978 19921750

[B199] Shieh D.-E. Chen Y.-Y. Yen M.-H. Chiang L.-C. Lin C.-C. (2004). Emodin-induced Apoptosis through P53-dependent Pathway in Human Hepatoma Cells. Life Sci. 74 (18), 2279–2290. 10.1016/j.lfs.2003.09.060 14987952

[B200] Shimpo K. Chihara T. Kaneko T. Beppu H. Wakamatsu K. Shinzato M. (2014). Inhibitory Effects of Low-Dose Aloe-Emodin on the Development of Colorectal Tumors in Min Mice. Asian Pac. J. Cancer Prev. 15 (14), 5587–5592. 10.7314/apjcp.2014.15.14.5587 25081669

[B201] Shivakumar S. Tadakaluru Y. L. Yakkanti R. R. R. Suresh S. R. Pasula C. S. (2017). Role of Quercetin in Chemoprevention against Wide Range of Carcinogens and Mutagens. Int. J. Drug Deliv. 9, 9. 10.5138/09750215.2040

[B202] Shon Y.-H. Park S.-D. Nam K.-S. (2006). Effective Chemopreventive Activity of Genistein against Human Breast Cancer Cells. BMB Rep. 39 (4), 448–451. 10.5483/bmbrep.2006.39.4.448 16889690

[B203] Shukla Y. Singh M. (2007). Cancer Preventive Properties of Ginger: a Brief Review. Food Chem. Toxicol. 45 (5), 683–690. 10.1016/j.fct.2006.11.002 17175086

[B204] Silva dos Santos J. Goncalves Cirino J. P. de Oliveira Carvalho P. Ortega M. M. (2021). The Pharmacological Action of Kaempferol in Central Nervous System Diseases: A Review. Front. Pharmacol. 11, 2143. 10.3389/fphar.2020.565700 PMC7838523 33519431

[B205] Song H. Bao J. Wei Y. Chen Y. Mao X. Li J. (2015). Kaempferol Inhibits Gastric Cancer Tumor Growth: An *In Vitro* and *In Vivo* Study. Oncol. Rep. 33 (2), 868–874. 10.3892/or.2014.3662 25500692

[B206] Srinivas G. Anto R. J. Srinivas P. Vidhyalakshmi S. Senan V. P. Karunagaran D. (2003). Emodin Induces Apoptosis of Human Cervical Cancer Cells through Poly (ADP-Ribose) Polymerase Cleavage and Activation of Caspase-9. Eur. J. Pharmacol. 473 (2-3), 117–125. 10.1016/s0014-2999(03)01976-9 12892828

[B207] Subramaniam A. Shanmugam M. K. Ong T. H. Li F. Perumal E. Chen L. (2013). Emodin Inhibits Growth and Induces Apoptosis in an Orthotopic Hepatocellular Carcinoma Model by Blocking Activation of STAT3. Br. J. Pharmacol. 170 (4), 807–821. 10.1111/bph.12302 23848338 PMC3799595

[B208] Subramaniam D. Ponnurangam S. Ramamoorthy P. Standing D. Battafarano R. J. Anant S. (2012). Curcumin Induces Cell Death in Esophageal Cancer Cells through Modulating Notch Signaling. PloS one 7 (2), e30590. 10.1371/journal.pone.0030590 22363450 PMC3281833

[B209] Sui J.-Q. Xie K.-P. Zou W. Xie M.-J. (2014). Emodin Inhibits Breast Cancer Cell Proliferation through the ERα-MAPK/Akt-Cyclin D1/Bcl-2 Signaling Pathway. Asian Pac. J. Cancer Prev. 15 (15), 6247–6251. 10.7314/apjcp.2014.15.15.6247 25124606

[B210] Sun X. Fu P. Xie L. Chai S. Xu Q. Zeng L. (2021). Resveratrol Inhibits the Progression of Cervical Cancer by Suppressing the Transcription and Expression of HPV E6 and E7 Genes. Int. J. Mol. Med. 47 (1), 335–345. 10.3892/ijmm.2020.4789 33236130 PMC7723400

[B211] Sun Y. Ren J. Wang F. (2021). [6]‐Gingerol Impedes 7, 12‐dimethylbenz (A) Anthracene‐induced Inflammation and Cell Proliferation‐associated Hamster Buccal Pouch Carcinogenesis through Modulating Nrf2 Signaling Events. J. Biochem. Mol. Toxicol. 35 (4), e22689. 10.1002/jbt.22689 33347680

[B212] Sun Z.-J. Chen G. Hu X. Zhang W. Liu Y. Zhu L.-X. (2010). Activation of PI3K/Akt/IKK-Α/nf-Κb Signaling Pathway Is Required for the Apoptosis-Evasion in Human Salivary Adenoid Cystic Carcinoma: its Inhibition by Quercetin. Apoptosis 15 (7), 850–863. 10.1007/s10495-010-0497-5 20386985

[B213] Sung H. Ferlay J. Siegel R. L. Laversanne M. Soerjomataram I. Jemal A. (2021). Global Cancer Statistics 2020: GLOBOCAN Estimates of Incidence and Mortality Worldwide for 36 Cancers in 185 Countries. CA: a Cancer J. clinicians 71 (3), 209–249. 10.3322/caac.21660 33538338

[B214] Thompson I. M. Goodman P. J. Tangen C. M. Lucia M. S. Miller G. J. Ford L. G. (2003). The Influence of Finasteride on the Development of Prostate Cancer. New Engl. J. Med. 349 (3), 215–224. 10.1056/NEJMoa030660 12824459

[B215] Tino A. B. Chitcholtan K. Sykes P. H. Garrill A. (2016). Resveratrol and Acetyl-Resveratrol Modulate Activity of VEGF and IL-8 in Ovarian Cancer Cell Aggregates via Attenuation of the NF-Κb Protein. J. ovarian Res. 9 (1), 1–12. 10.1186/s13048-016-0293-0 27906095 PMC5134119

[B216] Toden S. Goel A. (2017). The Holy Grail of Curcumin and its Efficacy in Various Diseases: Is Bioavailability Truly a Big Concern? J. restorative Med. 6 (1), 27. 10.14200/jrm.2017.6.0101 PMC6424351 30899605

[B217] Tong H. Huang Z. Chen H. Zhou B. Liao Y. Wang Z. (2020). Emodin Reverses Gemcitabine Resistance of Pancreatic Cancer Cell Lines through Inhibition of IKKβ/NF-Κb Signaling Pathway. OncoTargets Ther. 13, 9839. 10.2147/OTT.S253691 PMC7537840 33061461

[B218] Tossavainen A. (2004). Global Use of Asbestos and the Incidence of Mesothelioma. Int. J. Occup. Environ. Health 10 (1), 22–25. 10.1179/oeh.2004.10.1.22 15070022

[B219] Vijayakurup V. Thulasidasan A. T. Retnakumari A. P. Nandan C. D. Somaraj J. Antony J. (2019). Chitosan Encapsulation Enhances the Bioavailability and Tissue Retention of Curcumin and Improves its Efficacy in Preventing B [a] P-Induced Lung Carcinogenesis. Cancer Prev. Res. 12 (4), 225–236. 10.1158/1940-6207.CAPR-18-0437 30760502

[B220] Vinod B. Antony J. Nair H. Puliyappadamba V. Saikia M. Narayanan S. S. (2013). Mechanistic Evaluation of the Signaling Events Regulating Curcumin-Mediated Chemosensitization of Breast Cancer Cells to 5-fluorouracil. Cel Death Dis. 4 (2), e505. 10.1038/cddis.2013.26 PMC3734809 23429291

[B221] Vinod B. Nair H. Vijayakurup V. Shabna A. Shah S. Krishna A. (2015). Resveratrol Chemosensitizes HER-2-Overexpressing Breast Cancer Cells to Docetaxel Chemoresistance by Inhibiting Docetaxel-Mediated Activation of HER-2–Akt axis. Cel Death Discov. 1 (1), 1–9. 10.1038/cddiscovery.2015.61 PMC4979566 27551486

[B222] Vinod B. S. Maliekal T. T. Anto R. J. (2013). Phytochemicals as Chemosensitizers: from Molecular Mechanism to Clinical Significance. Antioxid. Redox signaling 18 (11), 1307–1348. 10.1089/ars.2012.4573 22871022

[B223] Wang G. Wang J. Chen X. Du S. Li D. Pei Z. (2013). The JAK2/STAT3 and Mitochondrial Pathways Are Essential for Quercetin Nanoliposome-Induced C6 Glioma Cell Death. Cel Death Dis. 4 (8), e746. 10.1038/cddis.2013.242 PMC3763427 23907460

[B224] Wang J. Li J. Cao N. Li Z. Han J. Li L. (2018). Resveratrol, an Activator of SIRT1, Induces Protective Autophagy in Non-small-cell Lung Cancer via Inhibiting Akt/mTOR and Activating P38-MAPK. OncoTargets Ther. 11, 7777. 10.2147/OTT.S159095 PMC6223384 30464525

[B225] Wang Y. James M. Wen W. Lu Y. Szabo E. Lubet R. A. (2010). Chemopreventive Effects of Pioglitazone on Chemically Induced Lung Carcinogenesis in Mice. Mol. Cancer Ther. 9 (11), 3074–3082. 10.1158/1535-7163.MCT-10-0510 21045137

[B226] Wang Y. Zhou P. Qin S. Xu D. Liu Y. Fu W. (2018). The Curcumin Analogs 2-pyridyl Cyclohexanone Induce Apoptosis via Inhibition of the JAK2–STAT3 Pathway in Human Esophageal Squamous Cell Carcinoma Cells. Front. Pharmacol. 9, 820. 10.3389/fphar.2018.00820 30186159 PMC6113578

[B227] Wang Z. Chen H. Chen J. Hong Z. Liao Y. Zhang Q. (2019). Emodin Sensitizes Human Pancreatic Cancer Cells to EGFR Inhibitor through Suppressing Stat3 Signaling Pathway. Cancer Manag. Res. 11, 8463. 10.2147/CMAR.S221877 31572001 PMC6756157

[B228] Way T.-D. Huang J.-T. Chou C.-H. Huang C.-H. Yang M.-H. Ho C.-T. (2014). Emodin Represses TWIST1-Induced Epithelial–Mesenchymal Transitions in Head and Neck Squamous Cell Carcinoma Cells by Inhibiting the β-catenin and Akt Pathways. Eur. J. Cancer 50 (2), 366–378. 10.1016/j.ejca.2013.09.025 24157255

[B229] Weinstock M. A. Thwin S. S. Siegel J. A. Marcolivio K. Means A. D. Leader N. F. (2018). Chemoprevention of Basal and Squamous Cell Carcinoma with a Single Course of Fluorouracil, 5%, Cream: a Randomized Clinical Trial. JAMA Dermatol. 154 (2), 167–174. 10.1001/jamadermatol.2017.3631 29299592 PMC5839275

[B230] Weisburger J. H. Dolan L. Pittman B. (1998). Inhibition of PhIP Mutagenicity by Caffeine, Lycopene, Daidzein, and Genistein. Mutat. Research/Genetic Toxicol. Environ. Mutagenesis 416, 125–128. 10.1016/s1383-5718(98)00083-7 9725998

[B231] Wolff J. E. Brown R. E. Buryanek J. Pfister S. Vats T. S. Rytting M. E. (2012). Preliminary Experience with Personalized and Targeted Therapy for Pediatric Brain Tumors. Pediatr. Blood Cancer 59 (1), 27–33. 10.1002/pbc.23402 22162424

[B232] Wu C.-C. Chen M.-S. Cheng Y.-J. Ko Y.-C. Lin S.-F. Chiu I.-M. (2019). Emodin Inhibits EBV Reactivation and Represses NPC Tumorigenesis. Cancers 11 (11), 1795. 10.3390/cancers11111795 PMC6896023 31731581

[B233] Xu D. Hu M.-J. Wang Y.-Q. Cui Y.-L. (2019). Antioxidant Activities of Quercetin and its Complexes for Medicinal Application. Molecules 24 (6), 1123. 10.3390/molecules24061123 PMC6470739 30901869

[B234] Yahyazadeh R. Baradaran Rahimi V. Yahyazadeh A. Mohajeri S. A. Askari V. R. (2021). Promising Effects of Gingerol against Toxins: A Review Article. BioFactors. 10.1002/biof.1779 34418196

[B235] Yang G. Nowsheen S. Aziz K. Georgakilas A. G. (2013). Toxicity and Adverse Effects of Tamoxifen and Other Anti-estrogen Drugs. Pharmacol. Ther. 139 (3), 392–404. 10.1016/j.pharmthera.2013.05.005 23711794

[B236] Yang S. Li X. Hu F. Li Y. Yang Y. Yan J. (2013). Discovery of Tryptanthrin Derivatives as Potent Inhibitors of Indoleamine 2, 3-dioxygenase with Therapeutic Activity in Lewis Lung Cancer (LLC) Tumor-Bearing Mice. J. Med. Chem. 56 (21), 8321–8331. 10.1021/jm401195n 24099220

[B237] Yu S.-T. Chen T.-M. Chern J.-W. Tseng S.-Y. Chen Y.-H. (2009). Downregulation of GSTπ Expression by Tryptanthrin Contributing to Sensitization of Doxorubicin-Resistant MCF-7 Cells through C-Jun NH2-terminal Kinase-Mediated Apoptosis. Anti-Cancer Drugs 20 (5), 382–388. 10.1097/CAD.0b013e32832a2cd4 19318911

[B238] Yu S.-T. Chen T.-M. Tseng S.-Y. Chen Y.-H. (2007). Tryptanthrin Inhibits MDR1 and Reverses Doxorubicin Resistance in Breast Cancer Cells. Biochem. biophysical Res. Commun. 358 (1), 79–84. 10.1016/j.bbrc.2007.04.107 17482571

[B239] Zan L. Chen Q. Zhang L. Li X. (2019). Epigallocatechin Gallate (EGCG) Suppresses Growth and Tumorigenicity in Breast Cancer Cells by Downregulation of miR-25. Bioengineered 10 (1), 374–382. 10.1080/21655979.2019.1657327 31431131 PMC6738446

[B240] Zeng L. Holly J. M. Perks C. M. (2014). Effects of Physiological Levels of the green tea Extract Epigallocatechin-3-Gallate on Breast Cancer Cells. Front. Endocrinol. 5, 61. 10.3389/fendo.2014.00061 PMC4019852 24847310

[B241] Zhang L. Chang C.-j. Bacus S. S. Hung M.-C. (1995). Suppressed Transformation and Induced Differentiation of HER-2/neu-Overexpressing Breast Cancer Cells by Emodin. Cancer Res. 55 (17), 3890–3896. 7543819

[B242] Zhang M. Zhao R. Wang D. Wang L. Zhang Q. Wei S. (2021). Ginger (Zingiber Officinale Rosc.) and its Bioactive Components Are Potential Resources for Health Beneficial Agents. Phytotherapy Res. 35 (2), 711–742. 10.1002/ptr.6858 32954562

[B243] Zhang W. Yin G. Dai J. Sun Y. Hoffman R. M. Yang Z. (2017). Chemoprevention by Quercetin of Oral Squamous Cell Carcinoma by Suppression of the NF-Κb Signaling Pathway in DMBA-Treated Hamsters. Anticancer Res. 37 (8), 4041–4049. 10.21873/anticanres.11789 28739686

[B244] Zhang X. Xia J. Zhang W. Luo Y. Sun W. Zhou W. (2017). Study on Pharmacokinetics and Tissue Distribution of Single Dose Oral Tryptanthrin in Kunming Mice by Validated Reversed-phase High-Performance Liquid Chromatography with Ultraviolet Detection. Integr. Med. Res. 6 (3), 269–279. 10.1016/j.imr.2017.05.001 28951841 PMC5605383

[B245] Zhang Y. Chen H. (2011). Genistein Attenuates WNT Signaling by Up-Regulating sFRP2 in a Human colon Cancer Cell Line. Exp. Biol. Med. 236 (6), 714–722. 10.1258/ebm.2011.010347 21571909

[B246] Zhong S. Jeong J.-H. Huang C. Chen X. Dickinson S. I. Dhillon J. (2021). Targeting INMT and Interrupting its Methylation Pathway for the Treatment of Castration Resistant Prostate Cancer. J. Exp. Clin. Cancer Res. 40 (1), 1–14. 10.1186/s13046-021-02109-z 34587977 PMC8482636

[B247] Zhou H.-B. Chen J.-J. Wang W.-X. Cai J.-T. Du Q. (2004). Apoptosis of Human Primary Gastric Carcinoma Cells Induced by Genistein. World J. Gastroenterol. WJG 10 (12), 1822. 10.3748/wjg.v10.i12.1822 15188515 PMC4572278

[B248] Zhou H.-B. Chen J.-M. Cai J.-T. Du Q. Wu C.-N. (2008). Anticancer Activity of Genistein on Implanted Tumor of Human SG7901 Cells in Nude Mice. World J. Gastroenterol. WJG 14 (4), 627. 10.3748/wjg.14.627 18203299 PMC2681158

[B249] Zhou P. Wang C. Hu Z. Chen W. Qi W. Li A. (2017). Genistein Induces Apoptosis of colon Cancer Cells by Reversal of Epithelial-To-Mesenchymal via a Notch1/NF-κB/slug/E-Cadherin Pathway. BMC cancer 17 (1), 1–10. 10.1186/s12885-017-3829-9 29202800 PMC5715491

[B250] Zhu W. Qin W. Zhang K. Rottinghaus G. E. Chen Y.-C. Kliethermes B. (2012). Trans-resveratrol Alters Mammary Promoter Hypermethylation in Women at Increased Risk for Breast Cancer. Nutr. Cancer 64 (3), 393–400. 10.1080/01635581.2012.654926 22332908 PMC3392022

[B251] Zhu X. Zhang X. Ma G. Yan J. Wang H. Yang Q. (2011). Transport Characteristics of Tryptanthrin and its Inhibitory Effect on P-Gp and MRP2 in Caco-2 Cells. J. Pharm. Pharm. Sci. 14 (3), 325–335. 10.18433/j3501w 21824448

